# Supplementary material for: Smoking and quit attempts during pregnancy and postpartum: a longitudinal UK cohort
Source: BMJ Open. 2017 Nov 15;7(11):e018746. doi: 10.1136/bmjopen-2017-018746 (PMC5695489; doi:10.1136/bmjopen-2017-018746)
Supplement: Supplementary file 1 [file bmjopen-2017-018746supp001.zip › Appendices/Pregnancy lifestyle survey baseline questionnaire Final Version 2_4 19 10 11 inc PIS v1.pdf]

The University of Nottingham
UNITED KINGDOM · CHINA · MALAYSIA

Nottingham University Hospitals NHS
NHS Trust

Researcher's use only

**Participant ID number / Initials**					/			
**Date returned**			/			/		
**Researcher's initials**								

Pregnancy lifestyle survey

BASELINE QUESTIONNAIRE
Final Version number: 2.4
Version date: 19th October 2011

Dear Madam,

We are looking at ways to improve the health and lifestyle of pregnant women and their babies. We are therefore inviting ALL women who come to the antenatal clinic to answer a few questions.

Your answers will be used in our research but are totally confidential and will only be seen by researchers from the University of Nottingham

Thank you for reading this and for your help.

Best wishes

Dr Tim Coleman
Division of Primary Care, University of Nottingham
QMC Medical School, Nottingham NG7 2RD

SCREENING QUESTIONS

Your answers to the questions on this page will determine whether you should complete the rest of the questionnaire. Please read the instructions carefully. Thank you.

S1 Have you completed this questionnaire before? (e.g. on a previous visit to the antenatal clinic or for a scan)

❑ Yes ❑ No ❑ Don't know

If you ticked **Yes**, please **hand back the questionnaire** - you are finished – thank you. Otherwise please continue

S2 Approximately, how many weeks pregnant are you?

[ ][ ] weeks ❑ Don't know

S3 How old are you?

[ ][ ] Years of age

S4 Please tick the box below next to the statement that best describes your smoking right now

1 ❑ I have never smoked (if you have ticked this box please hand back the questionnaire – you are finished)

2 ❑ I completely stopped smoking **more than** 3 months before finding out I was pregnant (if you have ticked this box please hand back the questionnaire – you are finished)

3 ❑ I completely stopped smoking at some time **in** the 3 months before finding out I was pregnant

4 ❑ I completely stopped smoking after I found out I was pregnant

5 ❑ I smoke occasionally, but not every day now I am pregnant

6 ❑ I smoke every day, but have cut down during my pregnancy

7 ❑ I smoke every day, about the same as before my pregnancy

8 ❑ I smoke every day, and I tend to smoke more than before my pregnancy

If you ticked one of the boxes 3-8, **and** you are between 8 and 26 weeks pregnant (or don't know), **and** are 16 years old or over, then please read the **NEXT PAGE**. Otherwise please **hand back the questionnaire** - you are finished – thank you.

(If you are not sure whether you should be filling in the rest of the questionnaire please ask the researcher)

Your answers mean that we are interested in finding out more about your views on smoking and pregnancy. We would be very grateful if you could read the following information that gives full details of the study.

If you want to ask any questions either before or after you read this then please ask to speak to our researcher who is in the clinic waiting area.

Once you have read this, if you are happy to continue, you can complete the longer questionnaire and once you have finished this we will give you a £5 gift voucher to thank you for your effort.

The University of Nottingham
UNITED KINGDOM · CHINA · MALAYSIA
Nottingham University Hospitals NHS
NHS Trust

INFORMATION ABOUT THE RESEARCH

Study title: *Pregnancy Lifestyle Survey*

Name of Researchers: Tim Coleman, Sue Cooper, Jo Leonardi-Bee, Jim Thornton, John Britton, Stephen Sutton, Felix Naughton, Michael Ussher and Kate Pickett

We are inviting you to take part in a questionnaire research study by the University of Nottingham. The information below is to help you understand why we are doing the research and what it involves. We will also answer any questions you have so that you can decide if you want to join the study.

What is the purpose of the study?
We want to find out about the times in their pregnancy that women smoke, when they might try to stop, plus your opinion on different types of support that could help with this. We also want to know how accurately GPs record when women smoke during their pregnancy.

Why have I been chosen?
We are asking you to take part because you have told us that either you smoke now, or you have smoked at some point since becoming pregnant or in the three months before this.

Do I have to take part?
No, this is entirely up to you. If you decide to take part then we will ask you to sign a consent form. Even if you sign this, you are free to leave the study at any time without giving a reason, and it would not affect the care you receive.

What will happen to me if I take part? What will I have to do?
There are three questionnaires altogether. There is one for you to complete today and then we will then send you two further questionnaires. We will send you the first of these when you are around 34 weeks pregnant and the second 3 months after

your baby is born. If we don't get them back then we will call you to ask if you would answer the questions over the phone. If you prefer, we can send them to you by email or you can fill them in on a special webpage. As we know that filling in the questionnaires takes some of your time, we will give you a £5 voucher for each one that you complete and return to us. If you take part in the study, a researcher will check your hospital antenatal records to ensure all is well with you and your baby before we send the questionnaires, but we will not use this information for any other reason.

As well as giving us your own contact details, we will also ask you for contact details of another family member. You don't have to give us these, but if you do we will also keep them strictly confidential and we will only use them to help us get in touch with you if you move or change your contact details.

Later, we want to see how accurately GPs record smoking in pregnancy. To do this we need your permission to look in your GP medical records to compare the information on smoking held there with what you tell us in the survey. We would keep your personal information for no longer than 7 years to allow us to do this (at the moment we don't have any funding to do this part of the study). You can indicate on the consent form if you are happy for us to do this.

Will my taking part in this study be kept confidential?
Yes. We will keep all information that is collected about you during the research strictly confidential. The questionnaires will not have your name and address on them, only a code. Only the researchers and people allowed to check that the research is carried out correctly could have access to your personal information.

After this study has started, researchers may think up important research questions that you could help them to answer. Therefore, we would like you to agree to allow other researchers based at the University of Nottingham to contact you to ask for your help with other studies. They would not do this unless a research ethics committee agreed to let them. You could ignore any contact letter about other research projects if you are not interested in taking part. You can indicate on the consent form if you are happy with this.

What are the possible disadvantages and risks of taking part?
Filling in the questionnaires will take a little of your time. Each one should take about 10 or 20 minutes to fill in.

What are the possible benefits of taking part?
We cannot promise the study will help you but the information we get from this study may help us to improve the help offered to women who want to try to stop smoking when they are pregnant. If, during the study, you decide that you want help to stop smoking, please tell us and we will let you know how you can receive NHS support with this.

What will happen if I don't want to carry on with the study?
Although we would like you to return the questionnaires, whether or not you do so is up to you. If you change your mind and don't want us to send you any more questionnaires, you can let us know by phone, text or email, or you can return the Freepost card we will give you. However, we wouldn't be able to erase the

information you had already given us and we may still use this in the study. This does not affect your legal rights.

What will happen to the results of the research study?
We will publish the study results but you will not be identified in any report. Findings will help the NHS decide how and when to offer smoking cessation support in pregnancy. You will be able to see a summary of study findings on the UK Centre for Tobacco Control Studies website at: http://www.nottingham.ac.uk/ukctcs/index.aspx

Who is organising and funding the research?
NHS National Institute for Health Research is funding the research. It is organised by the University of Nottingham.

Who has reviewed the research?
To protect your interests, an independent group of people, called a Research Ethics Committee, looks at all research in the NHS. Derbyshire Research Ethics Proportionate Review Sub-Committee have reviewed and approved this study.

What if there is a problem or you need further information
If you have any concerns about the study then please speak to the researchers or contact the project manager (contact details below). If you remain unhappy and wish to complain formally, you can do this by contacting NHS Complaints. Details can be obtained from your hospital.

You can also contact the researchers below if you need more information or would like to give feedback.

Dr Sue Cooper
Smoking in Pregnancy Project Manager
Tel 0115 823 1898
Email sue.cooper@nottingham.ac.uk

Dr Tim Coleman (*Chief Investigator*)
Tel 0115 823 0204
Email tim.coleman@nottingham.ac.uk

Katharine Bowker *(Research Fellow)*
Tel 0115 7484040
Email katharine.bowker@nottingham.ac.uk

Sophie Orton (*Research Fellow*)
Tel 0115 7484043
Email sophie.orton@nottingham.ac.uk

Smoking and Pregnancy Research Office
Division of Primary Care,
Room 1406, Tower Building
University of Nottingham
NG7 2RD

This information sheet is for you to keep.

Thank you very much for your time and help – we are very grateful for this.

The University of Nottingham
UNITED KINGDOM · CHINA · MALAYSIA

Nottingham University Hospitals NHS
NHS Trust

Researcher's use only

**Participant ID number / Initials**	_ _ _ _ / _ _ _ _
**Date returned**	_ _ / _ _ / _ _
**Researcher's initials**	_ _

Pregnancy lifestyle survey

YOUR HEALTH AND YOUR PREGNANCY

A1 How would you describe your health generally?

1 ❑ Excellent
2 ❑ Good
3 ❑ Fair
4 ❑ Poor

A2 Do you have a long standing physical or mental illness or disability? By longstanding I mean anything that has troubled you over a period of time or that is likely to affect you for some time into the future?

❑ Yes ❑ No

If Yes, does this illness or disability limit your activities in any ways?

❑ Yes ❑ No

A3 During the past month, have you often been bothered by feeling down, depressed or hopeless?

❑ Yes ❑ No

A4 During the past month, have you often been bothered by having little interest or pleasure in doing things?

❑ Yes ❑ No

A5 Please answer each of the following questions by circling the appropriate number. **Please circle one number per question.**

	Never	Almost never	Sometimes	Fairly often	Very often
In the last month, how often have you felt…					
…that you were unable to control the important things in your life?	1	2	3	4	5
…confident about your ability to handle your personal problems?	1	2	3	4	5
…that things were going your way?	1	2	3	4	5
…difficulties were piling up so high that you could not overcome them?	1	2	3	4	5

A6 Have you been pregnant before?

❑ Yes ❑ No

If Yes, did you smoke at all after finding out you were pregnant during your last pregnancy?

❑ Yes ❑ No ❑ I don't remember

A7 This time, were you planning to get pregnant or was it a surprise?

❑ Planning ❑ Was a surprise

YOUR SMOKING BEHAVIOUR AND BELIEFS

B1 When did you last smoke any cigarettes or tobacco (even a puff)?

- ❑ In the last 24 hours
- ❑ 1-6 days ago
- ❑ 7-30 days ago
- ❑ 1-2 months ago
- ❑ 2-3 months ago
- ❑ More than 3 months ago

B2 If you have a partner, do they smoke tobacco?

- ❑ Yes
- ❑ No
- ❑ I don't have a partner

B3 Does anyone who lives with you smoke tobacco in the home?

- ❑ Yes
- ❑ No

B4 How much of the time have you felt the urge to smoke in the past 24 hours?

- ❑ Not at all
- ❑ A little of the time
- ❑ Some of the time
- ❑ A lot of the time
- ❑ Almost all of the time
- ❑ All the time
- ❑ Don't know

B5 How strong have the urges been in the past 24 hours?

- ❑ No urges
- ❑ Slight
- ❑ Moderate
- ❑ Strong
- ❑ Very strong
- ❑ Extremely strong
- ❑ Don't know

B6 If you are planning on stopping smoking, or have already stopped, how long do you intend to stop for?

- ❑ Permanently/for good
- ❑ Until the birth of your baby/babies
- ❑ Unsure
- ❑ I am not planning on stopping smoking

B7 Please answer each of the following questions by circling the appropriate number. **Please circle one number per question.**

	**Not at all**	**A little**	**Moderately**	**Very much**	**Extremely**
How **determined** are you to stop smoking until your baby is born?	1	2	3	4	5
How **confident** are you that you can stop smoking until your baby is born?	1	2	3	4	5
How **determined** are you to stop smoking for good?	1	2	3	4	5
How **confident** are you that you can stop smoking for good?	1	2	3	4	5
How **confident** are you that you can stop smoking/remain stopped on your own (i.e. without help from a health professional)?	1	2	3	4	5
How **confident** are you that you can stop smoking/remain stopped with help from a health professional?	1	2	3	4	5

B8 Please indicate how much you agree with each statement below. **Please circle one number per question.**

	**Not at all**	**A little**	**Moderately**	**Very much**	**Extremely**
Smoking during pregnancy can cause serious harm to my baby	1	2	3	4	5
Smoking in pregnancy makes me feel uncomfortable or embarrassed	1	2	3	4	5
People I know continued to smoke when they were pregnant	1	2	3	4	5
I have support from my family or friends to help me stop smoking	1	2	3	4	5
People who are important to me think I should avoid smoking	1	2	3	4	5
Asking for professional support to help me stop smoking in pregnancy would make me feel uncomfortable or embarrassed	1	2	3	4	5

If you **SMOKE EVERY NOW & AGAIN or MORE OFTEN** continue to question C1 on the next page.

If you **DO NOT SMOKE AT THE MOMENT** go to question D1 on page 9

YOUR CURRENT SMOKING BEHAVIOUR

Please complete this section if you **SMOKE EVERY NOW & AGAIN or MORE OFTEN THAN THIS**

C1 Approximately how many cigarettes do you smoke each day?

- ❑ 0-5
- ❑ 6-10
- ❑ 11-15
- ❑ 16-20
- ❑ 21-30
- ❑ 31 or more

C2 How soon after waking do you smoke your first cigarette of the day?

- ❑ Within 5 minutes
- ❑ 6-30 minutes
- ❑ 31-60 minutes
- ❑ After 60 minutes

C3 Since finding out you were pregnant, have you tried to stop smoking?

- ❑ Yes
- ❑ No

If yes, please write in how many times you managed to stop smoking ***completely*** for at least 24 hours

[ ] times

C4 Since you found out you were pregnant, what was the longest you managed to remain stopped?

- ❑ I have not tried to stop smoking
- ❑ Less than 24 hours
- ❑ 1-6 days
- ❑ 7-30 days
- ❑ 1-2 months
- ❑ 2-3 months
- ❑ More than 3 months

C5 Are you seriously planning to quit?:

- ❑ Within the next 2 weeks
- ❑ Within the next 30 days
- ❑ Within the next 3 months
- ❑ No I am not seriously planning to quit

YOUR INTEREST IN GETTING HELP TO STOP SMOKING

All respondents should complete this section

D1 Since you found out you were pregnant, have you tried any of the following to help you stop smoking? (**Please tick all that apply**)

- ❑ Talked to your GP or a nurse about giving up smoking
- ❑ Talked to your midwife about giving up smoking
- ❑ Attended a NHS stop smoking service group session
- ❑ Attended a solo/individual NHS stop smoking service session (i.e. not with other people)
- ❑ Called a stop smoking telephone helpline
- ❑ Used Nicotine Replacement Therapy (e.g. nicotine patches or gum)
- ❑ Set a quit date
- ❑ Other. Please state: ______________________________
- ❑ None of the above

D2 Currently, how interested are you in receiving help with stopping smoking?

- ❑ Not at all
- ❑ A little
- ❑ Moderately
- ❑ Very much
- ❑ Extremely

D3 How **interested** would you be in the following types of help to stop smoking/stay stopped? Please answer by circling the appropriate number. **Please circle one number per question.**

	Not at all	A little	Moderately	Very much	Extremely
How interested would you be in stop-smoking help ***from a health professional*** *who offered you…*					
…a telephone helpline	1	2	3	4	5
…group sessions	1	2	3	4	5
…one-to-one sessions	1	2	3	4	5
How interested would you be in stop-smoking help ***that you can work through on your own (self-help)*** *if we gave you…*					
…a booklet	1	2	3	4	5
…a DVD	1	2	3	4	5
…a website	1	2	3	4	5
… text messages	1	2	3	4	5
…email	1	2	3	4	5
…an application on your mobile phone/device	1	2	3	4	5

D4 Please answer each of the following questions by circling the appropriate number. **Please circle one number per question.**

	Not at all	A little	Moderately	Very much	Extremely
*How **useful** do you think the following ways would be to help you to stop smoking/stay stopped?*					
A telephone helpline	1	2	3	4	5
Group sessions with a health professional	1	2	3	4	5
One-to-one sessions with a health professional	1	2	3	4	5
A self-help booklet	1	2	3	4	5
A DVD	1	2	3	4	5
A self-help website	1	2	3	4	5
Self-help mobile phone text messages	1	2	3	4	5
Self-help emails	1	2	3	4	5
A self-help application on your mobile phone/device	1	2	3	4	5
*If it were available, how **difficult** do you think it would be for you to **use** the following types of stop-smoking help?*					
A telephone helpline	1	2	3	4	5
Group sessions with a health professional	1	2	3	4	5
One-to-one sessions with a health professional	1	2	3	4	5
A self-help booklet	1	2	3	4	5
A DVD	1	2	3	4	5
A self-help website	1	2	3	4	5
Self-help mobile phone text messages	1	2	3	4	5
Self-help emails	1	2	3	4	5
A self-help application on your mobile phone/device	1	2	3	4	5

D5 Do any of the following describe your feelings about stop-smoking help that you work through on your own (ie, self-help)? **Please tick all that apply**

- ❑ I would miss having personal contact with a health professional
- ❑ It is too much effort to work through this type of support on my own
- ❑ It would be too difficult for me to understand this type of support
- ❑ I don't have the time to work through this type of support on my own
- ❑ I don't think this type of support would be much help with quitting smoking
- ❑ I think this type of support would be boring
- ❑ I would not read/work through this type of support if I received it
- ❑ I prefer to receive support from a health professional
- ❑ None of the above

D6 Which of the following applies to you? (**Please select one option per line**)

I own a mobile phone	❑ Yes	❑ No	❑ Don't know
I get free text messages as part of my tariff/package	❑ Yes	❑ No	❑ Don't know
I can install applications on my mobile phone	❑ Yes	❑ No	❑ Don't know
I have regular access to the internet/emails via my mobile phone	❑ Yes	❑ No	❑ Don't know
I have regular access to the internet/emails via a computer	❑ Yes	❑ No	❑ Don't know
I have an email account	❑ Yes	❑ No	❑ Don't know
I have a DVD player	❑ Yes	❑ No	❑ Don't know

ABOUT YOU

E1 At what age did you leave/finish full time education?

[ ][ ] Years of age ❑ I am still in education

E2 Which of the following qualifications do you have?

- ❑ None
- ❑ GCSEs or similar (e.g. level 1 diploma or NVQ level 1/2)
- ❑ A-levels/AS-levels or similar (e.g. level 2 diploma or NVQ level 3)
- ❑ Degree or similar (e.g. diploma in higher education or NVQ level 4)
- ❑ Other

E3 Do you own or rent your home?

❑ Own ❑ Rent ❑ Other

E4 Are there any cars or vans available for use in your household?

❑ Yes ❑ No

E5 Which of the following applies to you? **(Please tick all that apply)**

- ❑ I am in paid work at the moment
- ❑ I'm unemployed
- ❑ I'm a full-time student
- ❑ I'm a homemaker/full time parent
- ❑ Other

E6 Please describe your current or most recent paid job. Please leave blank if you have not previously been in paid work.

____

E7 Is your usual occupation manual or non-manual?

❑ Manual ❑ Non-manual ❑ Don't know ❑ Not applicable

E8 How would you describe your ethnic group?

❑ White - British
❑ White - Irish
❑ Other white background
❑ Mixed - white and black Caribbean
❑ Mixed - white and Asian
❑ Other mixed background
❑ Asian or Asian British - Indian
❑ Asian or Asian British - Pakistani
❑ Asian or Asian British - Bangladeshi
❑ Other Asian background
❑ Black or black British - Caribbean
❑ Black or black British - African
❑ Other black background
❑ Chinese
❑ Other. Please specify: ______________________________

Thank you for completing the questionnaire.

Please hand this to our researcher in clinic who will give you a £5 voucher and may talk with you about the rest of the study.

If you can't find the researcher, then please speak to one of the reception staff in clinic who will, if necessary, take your contact details so that the researcher can get in touch with you later.

If you haven't had time to complete the entire questionnaire today, or if you want some more time to think about it, then please ask the researcher or reception staff for a return envelope. Once you have decided, you can return the questionnaire along with your completed details on the attached sheet, and the researcher will contact you.

Thank you for your time today. We are very grateful for your help.

The University of Nottingham
UNITED KINGDOM · CHINA · MALAYSIA

Nottingham University Hospitals NHS
NHS Trust

Pregnancy lifestyle survey

CONTACT DETAILS

If you would like to take part in the study or you would to discuss this further, then please complete your personal details below.
The researcher will contact you as soon as possible

Name		
Address		
Telephone	Day:	Evening:
	Mobile:	
Best time to contact		
Email address		
