# Supplementary material for: Smoking and quit attempts during pregnancy and postpartum: a longitudinal UK cohort
Source: BMJ Open. 2017 Nov 15;7(11):e018746. doi: 10.1136/bmjopen-2017-018746 (PMC5695489; doi:10.1136/bmjopen-2017-018746)
Supplement: Supplementary file 1 [file bmjopen-2017-018746supp001.zip › Appendices/Pregnancy lifestyle survey Follow up questionnaire 1 Final Version 2_3 19 10 11.pdf]

Researcher's use only

Participant ID number / Initials					/			
Date returned			/			/		
Researcher's initials								

Pregnancy lifestyle survey

FOLLOW UP QUESTIONNAIRE 1
Final Version number: 2.3
Version date: 19th October 2011

Please complete this questionnaire **within the next two weeks** and then return it in the envelope provided (no stamp required).

The information you give us will be confidential and only used by the Pregnancy Lifestyle Survey researchers.

If you have any questions or concerns about this questionnaire, please call the Smoking and Pregnancy Research Office on 0115 823 1899.

Thank you for your help

Today's date: ....

YOUR SMOKING BEHAVIOUR AND BELIEFS

A1 Please tick the box below next to the statement that best describes your smoking right now

- ❑ I don’t smoke at all
- ❑ I smoke occasionally, but not every day
- ❑ I smoke every day, but have cut down during my pregnancy
- ❑ I smoke every day, about the same as before my pregnancy
- ❑ I smoke every day, and I tend to smoke more than before my pregnancy

A2 If you have a partner, do they smoke tobacco?

❑ Yes ❑ No ❑ I don't have a partner

A3 Does anyone who lives with you smoke tobacco in the home?

❑ Yes ❑ No

A4 How much of the time have you felt the urge to smoke in the past 24 hours?

- ❑ Not at all
- ❑ A little of the time
- ❑ Some of the time
- ❑ A lot of the time
- ❑ Almost all of the time
- ❑ All the time
- ❑ Don't know

A5 How strong have the urges been in the past 24 hours?

- ❑ No urges
- ❑ Slight
- ❑ Moderate
- ❑ Strong
- ❑ Very strong
- ❑ Extremely strong
- ❑ Don't know

A6 If you are planning on stopping smoking, or have already stopped, how long do you intend to stop for?

- ☐ Permanently/for good
- ☐ Until the birth of your baby/babies
- ☐ Unsure
- ☐ I am not planning on stopping smoking

A7 Please answer each of the following questions by circling the appropriate number. **Please circle one number per question.**

	**Not at all**	**A little**	**Moderately**	**Very much**	**Extremely**
How **determined** are you to stop smoking until your baby is born?	1	2	3	4	5
How **confident** are you that you can stop smoking until your baby is born?	1	2	3	4	5
How **determined** are you to stop smoking for good?	1	2	3	4	5
How **confident** are you that you can stop smoking for good?	1	2	3	4	5
How **confident** are you that you can stop smoking/remain stopped on your own (i.e. without help from a health professional)?	1	2	3	4	5
How **confident** are you that you can stop smoking/remain stopped with help from a health professional?	1	2	3	4	5

A8 Please indicate how much you agree with each statement below. **Please circle one number per question.**

	**Not at all**	**A little**	**Moderately**	**Very much**	**Extremely**
Smoking during pregnancy can cause serious harm to my baby	1	2	3	4	5
Smoking in pregnancy makes me feel uncomfortable or embarrassed	1	2	3	4	5
If I breathe in other people's smoke regularly it can seriously harm my unborn baby	1	2	3	4	5
People I know continued to smoke when they were pregnant	1	2	3	4	5
I have support from my family or friends to help me stop smoking	1	2	3	4	5
People who are important to me think I should avoid smoking	1	2	3	4	5
Asking for professional support to help me stop smoking in pregnancy would make me feel uncomfortable or embarrassed	1	2	3	4	5

A9 How concerned are you about putting on weight as a result of stopping smoking?

- ❑ Not at all
- ❑ A little
- ❑ Moderately
- ❑ Very much
- ❑ Extremely

If you **SMOKE EVERY NOW & AGAIN or MORE OFTEN** continue to question B1 on the next page. If you **DO NOT SMOKE AT THE MOMENT** go to C1 on page 6

YOUR CURRENT SMOKING BEHAVIOUR

Please complete this section if you **SMOKE EVERY NOW & AGAIN or MORE OFTEN THAN THIS**

B1 Approximately how many cigarettes do you smoke each day?

- ❑ 0-5
- ❑ 6-10
- ❑ 11-15
- ❑ 16-20
- ❑ 21-30
- ❑ 31 or more

B2 How soon after waking do you smoke your first cigarette of the day?

- ❑ Within 5 minutes
- ❑ 6-30 minutes
- ❑ 31-60 minutes
- ❑ After 60 minutes

B3 Since completing the first study questionnaire, have you tried to stop smoking?

- ❑ Yes
- ❑ No

If yes, please write in how many times during this period you managed to stop smoking ***completely*** for at least 24 hours

☐ times

B4 Are you seriously planning to quit?:

- ❑ Within the next 2 weeks
- ❑ Within the next 30 days
- ❑ Within the next 3 months
- ❑ No I am not seriously planning to quit

YOUR INTEREST IN GETTING HELP TO STOP SMOKING

All respondents should complete this section

C1 Since you completed the first study questionnaire, have you tried any of the following to help you stop smoking? (**Please tick all that apply**)

- ❑ Talked to your GP or a nurse about giving up smoking
- ❑ Talked to your midwife about giving up smoking
- ❑ Attended a NHS stop smoking service group session
- ❑ Attended a solo/individual NHS stop smoking service session (i.e. not with other people)
- ❑ Called a stop smoking telephone helpline
- ❑ Used Nicotine Replacement Therapy (e.g. nicotine patches or gum)
- ❑ Set a quit date
- ❑ Other. Please state: ______________________________
- ❑ None of the above

C2 Currently, how interested are you in receiving help with stopping smoking?

- ❑ Not at all
- ❑ A little
- ❑ Moderately
- ❑ Very much
- ❑ Extremely

C3 How **interested** would you be in the following types of help to stop smoking/stay stopped? Please answer by circling the appropriate number. **Please circle one number per question.**

	**Not at all**	**A little**	**Moderately**	**Very much**	**Extremely**
How interested would you be in stop-smoking help ***from a health professional*** *who offered you…*					
…a telephone helpline	1	2	3	4	5
…group sessions	1	2	3	4	5
…one-to-one sessions	1	2	3	4	5
How interested would you be in stop-smoking help ***that you can work through on your own (self-help)*** *if we gave you…*					
…a booklet	1	2	3	4	5
…a DVD	1	2	3	4	5
…a website	1	2	3	4	5
… text messages	1	2	3	4	5
…email	1	2	3	4	5
…an application on your mobile phone/device	1	2	3	4	5

C4 Please answer each of the following questions by circling the appropriate number. **Please circle one number per question.**

	**Not at all**	**A little**	**Moderately**	**Very much**	**Extremely**
*How **useful** do you think the ways would be to help you to stop smoking/stay stopped?*					
A telephone helpline	1	2	3	4	5
Group sessions with a health professional	1	2	3	4	5
One-to-one sessions with a health professional	1	2	3	4	5
A self-help booklet	1	2	3	4	5
A DVD	1	2	3	4	5
A self-help website	1	2	3	4	5
Self-help mobile phone text messages	1	2	3	4	5
Self-help emails	1	2	3	4	5
A self-help application on your mobile phone/device	1	2	3	4	5
*If it were available, how **difficult** do you think it would be for you to **use** the following types of stop-smoking help?*					
A telephone helpline	1	2	3	4	5
Group sessions with a health professional	1	2	3	4	5
One-to-one sessions with a health professional	1	2	3	4	5
A self-help booklet	1	2	3	4	5
A DVD	1	2	3	4	5
A self-help website	1	2	3	4	5
Self-help mobile phone text messages	1	2	3	4	5
Self-help emails	1	2	3	4	5
A self-help application on your mobile phone/device	1	2	3	4	5

C5 Do any of the following describe your feelings about stop-smoking help that you work through on your own (self-help)? **Please tick all that apply**

- ❑ I would miss having personal contact with a health professional
- ❑ It is too much effort to work through this type of support on my own
- ❑ It would be too difficult for me to understand this type of support
- ❑ I don’t have the time to work through this type of support on my own
- ❑ I don’t think this type of support would be much help with quitting smoking
- ❑ I think this type of support would be boring
- ❑ I would not read/work through this type of support if I received it
- ❑ I prefer to receive support from a health professional
- ❑ None of the above

Please turn over the page for the final section of questions →

YOUR HEALTH AND YOUR PREGNANCY

All respondents should complete this section

D1 During the past month, have you often been bothered by feeling down, depressed or hopeless?

❑ Yes ❑ No

D2 During the past month, have you often been bothered by having little interest or pleasure in doing things?

❑ Yes ❑ No

D3 Please answer each of the following questions by circling the appropriate number. **Please circle one number per question.**

	**Never**	**Almost never**	**Sometimes**	**Fairly often**	**Very often**
In the last month, how often have you felt…					
...that you were unable to control the important things in your life?	1	2	3	4	5
...confident about your ability to handle your personal problems?	1	2	3	4	5
...that things were going your way?	1	2	3	4	5
...difficulties were piling up so high that you could not overcome them?	1	2	3	4	5

D4 How much have you had any of the following **during your pregnancy**? **Please circle one number per question.**

	**Not at all**	**A little**	**Moderately**	**Very much**	**Extremely**
I have felt nauseous or sick	1	2	3	4	5
I have vomited	1	2	3	4	5

Thank you for completing the questionnaire

Please return **within the next 2 weeks** in the envelope provided (no stamp required)
