# Supplementary material for: Smoking and quit attempts during pregnancy and postpartum: a longitudinal UK cohort
Source: BMJ Open. 2017 Nov 15;7(11):e018746. doi: 10.1136/bmjopen-2017-018746 (PMC5695489; doi:10.1136/bmjopen-2017-018746)
Supplement: Supplementary file 1 [file bmjopen-2017-018746supp001.zip › Appendices/Pregnancy lifestyle survey Follow up questionnaire 2 Final Version 2_0 02 12 11.pdf]

The University of Nottingham
UNITED KINGDOM · CHINA · MALAYSIA

Researcher's use only

**Participant ID number / Initials**					/			
**Date returned**								
**Researcher's initials**								

Pregnancy lifestyle survey

FOLLOW UP QUESTIONNAIRE 2
Final Version number: 2.0
Version date: 2nd December 2011

Please complete this questionnaire **within the next two weeks** and then return it in the envelope provided (no stamp required).

The information you give us will be confidential and only used by the Pregnancy Lifestyle Survey researchers.

If you have any questions or concerns about this questionnaire, please call the Smoking and Pregnancy Research Office on 0115 823 1899.

Thank you for your help

Today's date: ....

YOUR SMOKING BEHAVIOUR AND BELIEFS

A1 Please tick the box below next to the statement that best describes your smoking right now

- ❑ I don’t smoke at all
- ❑ I smoke occasionally, but not every day
- ❑ I smoke every day, but less than when I was pregnant
- ❑ I smoke every day, and about the same as when I was pregnant
- ❑ I smoke every day, and I tend to smoke more now than when I was pregnant

A2 Did you smoke at all in the week before the birth of your baby ?

❑ Yes ❑ No ❑ I can’t remember

A3 Have you smoked at all since the birth of your baby?

❑ Yes ❑ No (If No – go to question A4)

If Yes, how soon after the birth of your baby did you first smoke?

- ❑ Within 24 hours
- ❑ 1-6 days
- ❑ 7-30 days
- ❑ 1-2 months
- ❑ More than 2 months

A4 If you have a partner, do they smoke tobacco?

❑ Yes ❑ No ❑ I don't have a partner

A5 How much of the time have you felt the urge to smoke in the past 24 hours?

- ❑ Not at all
- ❑ A little of the time
- ❑ Some of the time
- ❑ A lot of the time
- ❑ Almost all of the time
- ❑ All the time
- ❑ Don't know

A6 How strong have the urges been in the past 24 hours?

- ❑ No urges
- ❑ Slight
- ❑ Moderate
- ❑ Strong
- ❑ Very strong
- ❑ Extremely strong
- ❑ Don't know

A7 Please answer each of the following questions by circling the appropriate number. **Please circle one number per question.**

	Not at all	A little	Moderately	Very much	Extremely
How **determined** are you to stop smoking for good?	1	2	3	4	5
How **confident** are you that you can stop smoking for good?	1	2	3	4	5
How **confident** are you that you can stop smoking/remain stopped on your own (i.e. **without** help from a health professional)?	1	2	3	4	5
How **confident** are you that you can stop smoking/remain stopped **with** help from a health professional?	1	2	3	4	5
Do you have support from family or friends to help you stop smoking?	1	2	3	4	5
Do people who are important to you think you should avoid smoking?	1	2	3	4	5

A8 How concerned are you about putting on weight as a result of stopping smoking?

- ❑ Not at all
- ❑ A little
- ❑ Moderately
- ❑ Very much
- ❑ Extremely

If you **SMOKE EVERY NOW & AGAIN or MORE OFTEN** continue to question B1 on the next page. If you **DO NOT SMOKE AT THE MOMENT** go to C1 on page 5

YOUR CURRENT SMOKING BEHAVIOUR

Please complete this section if you **SMOKE EVERY NOW AND AGAIN or MORE OFTEN THAN THIS**

B1 Approximately how many cigarettes do you smoke each day?

- ❑ 0-5
- ❑ 6-10
- ❑ 11-15
- ❑ 16-20
- ❑ 21-30
- ❑ 31 or more

B2 How soon after waking do you smoke your first cigarette of the day?

- ❑ Within 5 minutes
- ❑ 6-30 minutes
- ❑ 31-60 minutes
- ❑ After 60 minutes

B3 Since the birth of your baby have you tried to stop smoking?

- ❑ Yes
- ❑ No

If yes, please write in how many times during this period you managed to stop smoking ***completely*** for at least 24 hours

☐ times

B4 Are you seriously planning to quit?

- ❑ Within the next 2 weeks
- ❑ Within the next 30 days
- ❑ Within the next 3 months
- ❑ No I am not seriously planning to quit

YOUR INTEREST IN GETTING HELP TO STOP SMOKING

All respondents should complete this section

C1 Since the birth of your baby, have you tried any of the following to help you stop smoking / remain stopped? (**Please tick all that apply**)

- ❑ Talked to your GP or a nurse about giving up smoking
- ❑ Talked to your midwife about giving up smoking
- ❑ Attended a NHS stop smoking service group session
- ❑ Attended a solo/individual NHS stop smoking service session (i.e. one-to-one - not with other people)
- ❑ Called a stop smoking telephone helpline
- ❑ Used Nicotine Replacement Therapy (e.g. nicotine patches or gum)
- ❑ Set a quit date
- ❑ Other. Please state: ____________________
- ❑ None of the above

C2 Currently, how interested are you in receiving help with stopping smoking?

- ❑ Not at all
- ❑ A little
- ❑ Moderately
- ❑ Very much
- ❑ Extremely

C3 How **interested** would you be in the following types of help to stop smoking/stay stopped? Please answer by circling the appropriate number. **Please circle one number per question.**

	Not at all	A little	Moderately	Very much	Extremely
How interested would you be in stop-smoking help ***from a health professional*** *who offered you…*					
**…**a telephone helpline	1	2	3	4	5
**…**group sessions	1	2	3	4	5
**…**one-to-one sessions	1	2	3	4	5
How interested would you be in stop-smoking help ***that you can work through on your own (self-help)*** *if we gave you…*					
**…**a booklet	1	2	3	4	5
…a DVD	1	2	3	4	5
**…**a website	1	2	3	4	5
**…** text messages	1	2	3	4	5
**…**email	1	2	3	4	5
**…**an application (app) on your mobile phone / device	1	2	3	4	5

C4 Please answer each of the following questions by circling the appropriate number. **Please circle one number per question.**

*How **USEFUL** do you think the following ways would be to help you to stop smoking/stay stopped?*					
	**Not at all**	**A little**	**Moderately**	**Very much**	**Extremely**
A telephone helpline	1	2	3	4	5
Group sessions with a health professional	1	2	3	4	5
One-to-one sessions with a health professional	1	2	3	4	5
A self-help booklet	1	2	3	4	5
A DVD	1	2	3	4	5
A self-help website	1	2	3	4	5
Self-help mobile phone text messages	1	2	3	4	5
Self-help emails	1	2	3	4	5
A self-help application (app) on your mobile phone / device	1	2	3	4	5

C5 Please answer each of the following questions by circling the appropriate number. **Please circle one number per question.**

*If it were available, how **DIFFICULT** do you think it would be for you to **use** the following types of stop-smoking help?*					
	**Not at all**	**A little**	**Moderately**	**Very much**	**Extremely**
A telephone helpline	1	2	3	4	5
Group sessions with a health professional	1	2	3	4	5
One-to-one sessions with a health professional	1	2	3	4	5
A self-help booklet	1	2	3	4	5
A DVD	1	2	3	4	5
A self-help website	1	2	3	4	5
Self-help mobile phone text messages	1	2	3	4	5
Self-help emails	1	2	3	4	5
A self-help application (app) on your mobile phone/device	1	2	3	4	5

C6 Do any of the following describe your feelings about stop-smoking help that you work through on your own (self-help)? **Please tick all that apply**

- ❑ I would miss having personal contact with a health professional
- ❑ It is too much effort to work through this type of support on my own
- ❑ It would be too difficult for me to understand this type of support
- ❑ I don’t have the time to work through this type of support on my own
- ❑ I don’t think this type of support would be much help with quitting smoking
- ❑ I think this type of support would be boring
- ❑ I would not read/work through this type of support if I received it
- ❑ I prefer to receive support from a health professional
- ❑ None of the above

YOUR HEALTH

All respondents should complete this section

D1 During the past month, have you often been bothered by feeling down, depressed or hopeless?

❑ Yes ❑ No

D2 During the past month, have you often been bothered by having little interest or pleasure in doing things?

❑ Yes ❑ No

D3 Please answer each of the following questions by circling the appropriate number. **Please circle one number per question.**

	**Never**	**Almost never**	**Sometimes**	**Fairly often**	**Very often**
In the last month, how often have you felt…					
**…**that you were unable to control the important things in your life?	1	2	3	4	5
**…**confident about your ability to handle your personal problems?	1	2	3	4	5
**…**that things were going your way?	1	2	3	4	5
**…**difficulties were piling up so high that you could not overcome them?	1	2	3	4	5

D4 Please answer each of the following questions by circling the appropriate number. **Please circle one number per question.**

	**Never**	**Almost never**	**Sometimes**	**Fairly often**	**Very often**
How often do you smoke in your home nowadays?	1	2	3	4	5
How often do other people smoke in your home nowadays?	1	2	3	4	5

D5 Please indicate how much you **agree** with each statement below. **Please circle one number per question.**

	**Not at all**	**A little**	**Moderately**	**Very much**	**Extremely**
If my baby regularly breathes in people's tobacco smoke, it can seriously harm him/her	1	2	3	4	5
Smoking in the home can seriously harm babies (under 1 year old)	1	2	3	4	5
Smoking in the home can seriously harm children (over 1 year old)	1	2	3	4	5
Smoking in the home but not in the same room as a baby can seriously harm him/her	1	2	3	4	5
Smoking in the home makes my house smell unpleasant	1	2	3	4	5

D6 How old was your baby when he/she last had breast milk?

- ❑ Never took breast milk
- ❑ Less than one week
- ❑ Less than one month
- ❑ Over one month but stopped breastfeeding
- ❑ Still breastfeeding

The next questions are about the different sorts of feelings parents might have when caring for young children. This includes their relationship with the baby, confidence in their parenting ability and their family routine. For each one please say which is closest to how **you** feel.

D7 When you are caring for your baby, how often do you feel annoyed or irritated?

- ❑ Almost all the time
- ❑ Very frequently
- ❑ Frequently
- ❑ Occasionally
- ❑ Rarely
- ❑ Never

D8 When you are caring for your baby, do you feel that you are…

- ❑ Very unskilled and lacking in confidence?
- ❑ Fairly unskilled and lacking in confidence?
- ❑ Fairly skilled and confident?
- ❑ Very skilled and confident?

D9 When you think about the things that you have had to give up because of your baby do you find that you…

- ❑ resent / dislike this a lot?
- ❑ resent / dislike this a fair amount?
- ❑ resent / dislike this a bit?
- ❑ don't resent / dislike this at all?

D10 How often do you have enough of the following things in your family? **Please circle one number per question.**

	**Never enough**	**Rarely enough**	**Sometimes enough**	**Usually enough**	**Almost always enough**
Money to pay monthly bills	1	2	3	4	5
Use of a car (either your own or someone else's)	1	2	3	4	5
Money to buy things for yourself	1	2	3	4	5
Money to save	1	2	3	4	5

D11 The following questions are about your day-to-day life and routines

Do you usually eat at least one meal a day at home with family or friends?	❑ Yes	❑ No
Do you have a diary or a calendar for keeping track of appointments?	❑ Yes	❑ No
Do you plan your spending money or make a budget for yourself?	❑ Yes	❑ No
Do you regularly care for anybody who has either a long-term illness or a problem with alcohol or drugs?	❑ Yes	❑ No
Do you have people living with you that you often wish weren't there?	❑ Yes	❑ No

Thank you for completing the questionnaire.
Please return **within the next 2 weeks** in the envelope provided (no stamp required)
