# Supplementary material for: Smoking and quit attempts during pregnancy and postpartum: a longitudinal UK cohort
Source: BMJ Open. 2017 Nov 15;7(11):e018746. doi: 10.1136/bmjopen-2017-018746 (PMC5695489; doi:10.1136/bmjopen-2017-018746)
Supplement: Supplementary file 2 [file bmjopen-2017-018746supp002.pdf]

Appendix Table S1 Cross-sectional data: participants' smoking behaviour for returned questionnaires only at each follow up point

**Characteristic**	**Baseline (Early pregnancy) N (%)**	**Late pregnancy N (%)**	**Postpartum N (%)**
**Respondents** (response rate)	850	509 (59.9)	476 (56.0)
**Smoking status**			
Current smoker	488 (57.4)	252 (49.5)	281 (59.0)
Recent ex-smoker	362 (42.6)	257 (50.5)	195 (41.0)
**Current smoking behaviour (all participants)**			
Stopped smoking during 3 months prior to pregnancy	61 (7.2)		
Stopped smoking after learning of pregnancy/don't smoke at all now	301 (35.4)	257 (50.5)	195 (41.0)
Smoke occasionally, but not every day now pregnant	117 (13.8)	61 (12.0)	84 (17.6)
Smoke every day, but have cut down since: learning of pregnancy/during pregnancy	304 (35.8)	144 (28.3)	56 (11.8)
Smoke every day, about the same as before/during pregnancy	64 (7.5)	42 (8.3)	80 (16.8)
Smoke every day, tend to smoke more than before/during pregnancy	3 (0.4)	4 (0.8)	59 (12.4)
Missing	0	1 (0.2)	2 (0.4)
**Current smokers only:**			
**Reported quit attempt since learning of pregnancy/previous questionnaire/birth of baby** [a]			
Yes	272 (55.7)	130 (51.6)	78 (27.8)
No	200 (41.0)	119 (47.2)	191 (68.0)
Missing	16 (3.3)	3 (1.2)	12 (4.3)
**If have made a quit attempt, attempt lasted at least 24 hours**			
Yes<br>(% of all current smokers)<br>(% of current smokers making a quit attempt)	178<br>(36.5%)<br>(65.4%)	103<br>(40.9%)<br>(79.2%)	61<br>(21.7%)<br>(78.2%)
No<br>(% of all current smokers)<br>(% of current smokers making a quit attempt)	90<br>(18.4%)<br>(33.1%)	19<br>(7.5%)<br>(14.6%)	6<br>(2.1%)<br>(7.7%)
**Number of quit attempts >24 hours, median (IQR)**	2 (1-3)	2 (1-5)	2 (1-4)
**Cigarettes per day**			
0-5	191 (39.1)	99 (39.3)	102 (36.3)
6-10	151 (30.9)	85 (33.7)	83 (29.5)
11-15	74 (15.2)	32 (12.7)	55 (19.6)
16-20	47 (9.6)	24 (9.5)	30 (10.7)
21-30	8 (1.6)	9 (3.6)	5 (1.8)
≥31	2 (0.4)	0 (0)	1 (0.4)
Missing	15 (3.1)	3 (1.2)	5 (1.8)
**Time to first cigarette**			
< 5 minutes	97 (19.9)	31 (12.3)	28 (10.0)
6-30 minutes	163 (33.4)	79 (31.3)	83 (29.5)
31-60 minutes	89 (18.2)	44 (17.5)	50 (17.8)

>60 minutes	117 (24.0)	91 (36.1)	105 (37.4)
Missing	22 (4.5)	7 (2.8)	15 (5.3)
**Heaviness of Smoking Index (HSI)**			
Low dependence (0-2)	310 (63.5)	178 (70.6)	189 (67.3)
Moderate dependence (3-4)	146 (29.9)	60 (23.8)	75 (26.7)
High dependence (5-6)	8 (1.6)	6 (2.4)	2 (0.7)
Missing	24 (4.9)	8 (3.2)	15 (5.3)
**Intention to quit smoking**			
Intending to quit within next 2 weeks	138 (28.3)	51 (20.2)	20 (7.1)
Intending to quit within next 30 days	59 (12.1)	23 (9.1)	22 (7.8)
Intending to quit within next 3 months	76 (15.6)	73 (29.0)	100 (35.6)
Not seriously intending to quit	176 (36.1)	90 (35.7)	123 (43.8)
Missing	39 (8.0)	15 (6.0)	16 (5.7)

[a] Quit attempts: at baseline, since finding out about the pregnancy; at follow-up 1, since completing previous survey; follow-up 2, since birth of baby
