# Supplementary material for: Smoking and quit attempts during pregnancy and postpartum: a longitudinal UK cohort
Source: BMJ Open. 2017 Nov 15;7(11):e018746. doi: 10.1136/bmjopen-2017-018746 (PMC5695489; doi:10.1136/bmjopen-2017-018746)
Supplement: Author's manuscript [file bmjopen-2017-018746.draft_revisions.pdf]

BMJ Open is committed to open peer review. As part of this commitment we make the peer review history of every article we publish publicly available.

When an article is published we post the peer reviewers' comments and the authors' responses online. We also post the versions of the paper that were used during peer review. These are the versions that the peer review comments apply to.

The versions of the paper that follow are the versions that were submitted during the peer review process. They are not the versions of record or the final published versions. They should not be cited or distributed as the published version of this manuscript.

BMJ Open is an open access journal and the full, final, typeset and author-corrected version of record of the manuscript is available on our site with no access controls, subscription charges or pay-per-view fees (http://bmjopen.bmj.com).

If you have any questions on BMJ Open's open peer review process please email editorial.bmjopen@bmj.com

BMJ Open

Patterns of smoking and quit attempts during pregnancy and postpartum: a longitudinal UK cohort

Journal:	*BMJ Open*
Manuscript ID	bmjopen-2017-018746
Article Type:	Research
Date Submitted by the Author:	18-Jul-2017
Complete List of Authors:	Cooper, Sue; University of Nottingham, Division of Primary Care<br>Orton, Sophie; University of Nottingham, Division of Primary Care<br>Leonardi-Bee, Jo; Uni Nottingham<br>Brotherton, Emma; University of Nottingham, Division of Primary Care<br>Vanderbloemen, Laura; Imperial College London, Department of Primary Care and Public Health<br>Bowker, Katharine; University of Nottingham, Division of Primary Care<br>Naughton, Felix; University of East Anglia Faculty of Medicine and Health Sciences, School of Health Sciences<br>Ussher, Michael; St George's University of London, Division of Population Health Sciences and Education<br>Pickett, Kate; University of York<br>Sutton, Stephen; University of Cambridge,<br>Coleman, Tim; University of Nottingham, Division of Primary Care
**Primary Subject Heading**:	Smoking and tobacco
Secondary Subject Heading:	Addiction, Epidemiology, Obstetrics and gynaecology, Public health
Keywords:	Smoking cessation, Pregnancy, Longitudinal research, Quit attempts, Postpartum relapse, Survey research

SCHOLARONE™
Manuscripts

Patterns of smoking and quit attempts during pregnancy and postpartum: a longitudinal UK cohort

Sue Cooper PhD[1] *, Sophie Orton PhD[1], Jo Leonardi-Bee PhD[2], Emma Brotherton BMedSci[1], Laura Vanderbloemen PhD[3], Katharine Bowker PhD[1], Felix Naughton PhD[4], Michael Ussher PhD[5], Kate E. Pickett PhD[6], Stephen Sutton PhD[7], Tim Coleman MD[1]

[1] Division of Primary Care, University of Nottingham, University Park, Nottingham, NG7 2RD. UK

[2] Division of Epidemiology and Public Health, University of Nottingham, Nottingham, UK

[3] Department of Primary Care and Public Health, Imperial College London, London, UK

[4] School of Health Sciences, University of East Anglia, Norwich, UK

[5] Population Health Research Institute, St. Georges, University of London, London, UK

[6] Department of Health Sciences, University of York, York, UK

[7] Behavioural Science Group, University of Cambridge, Cambridge, UK

* Correspondence to: sue.cooper@nottingham.ac.uk

Word Count 3847

For peer review only

ABSTRACT

Objectives

Pregnancy motivates women to try stopping smoking, but little is known about timing of their quit attempts and how quitting intentions change during pregnancy and postpartum. Using longitudinal data, this study aimed to document women's smoking and quitting behaviour throughout pregnancy and after delivery.

Design

Longitudinal cohort survey with questionnaires at baseline (8-26 weeks gestation), late pregnancy (34-36 weeks) and 3 months after delivery.

Setting

Two maternity hospitals in one National Health Service hospital trust, Nottingham, England.

Participants

850 pregnant women, aged 16 or over, who were current smokers or had smoked in the 3 months before pregnancy, were recruited between August 2011 and August 2012.

Outcome measures

Self-reported smoking behaviour, quit attempts and quitting intentions.

Results

Smoking rates, adjusting for non-response at follow-up, were 57.4% (95% CI 54.1-60.7) at baseline, 59.1% (95% CI 54.9-63.4) in late pregnancy and 67.1% (95% CI 62.7-71.5) 3 months postpartum. At baseline, 272 of 488 current smokers had tried to quit since becoming pregnant (55.7%, 95% CI 51.3-60.1); 51.3% (95% CI 44.7-58.0) tried quitting between baseline and late pregnancy and 27.4% (95% CI 21.7-33.2) after childbirth. The percentage who intended to quit within the next month fell as pregnancy

For peer review only

progressed, from 40.4% (95% CI 36.1-44.8) at baseline to 29.7% (95% CI 23.8-35.6) in late pregnancy and 14.2% (95% CI 10.0-18.3) postpartum. Postpartum relapse was lower among women who quit in the 3 months before pregnancy (17.8%, 95% CI 6.1-29.4) than those who stopped between baseline and late pregnancy (42.9%, 95% CI 24.6-61.3)

Conclusions

Many pregnant smokers make quit attempts throughout pregnancy and postpartum, but intention to quit reduces; there is no evidence that smoking rates fall during gestation.

Keywords

Smoking cessation, pregnancy, longitudinal research, quit attempts, postpartum relapse, survey research

For peer review only

Strengths and limitations of this study

- As far as we are aware, this is the only study to investigate timing of quit attempts and propensity to stop smoking during pregnancy and postpartum, and to quantify longitudinal changes.
- Smoking behaviour is self-reported rather than validated; misreporting due to recall bias may have been minimised by collecting data at three time points, and by there being no expectation that they should try to stop smoking.
- Later survey findings were adjusted using multiple imputation to help address non-response bias due to attrition.
- As the study was conducted in just one geographical area of the UK and participants were predominantly white British, findings might not be generalisable; however, the demographic profile of participants was similar to that of other UK cohorts of pregnant smokers.

For peer review only

INTRODUCTION

Smoking in pregnancy is associated with increased risks of miscarriage, stillbirth, prematurity, low birth weight, perinatal morbidity and mortality, neo-natal and sudden infant death, infant respiratory problems, poorer infant cognition, and adverse infant behavioural outcomes.[1 2] Internationally, large numbers of pregnant women smoke; with rates between 12% and 22% in high income countries[3-6] and rates increasing in emerging and developing economies.[7] Pregnancy is probably the event which most motivates female smokers to try quitting; for example, in the UK over 50 per cent of pregnant smokers try to stop[5] and pregnant women are, therefore, particularly likely to be interested in receiving cessation support. Some health systems systematically offer such support; in the UK this is largely done in early pregnancy,[8] although official guidance recommends that support is provided throughout gestation.[9]

We are aware of no studies that have investigated when, in pregnancy, smokers have the greatest propensity to try stopping, the timing of any quit attempts and potential influences on this. Women who smoke before pregnancy have varied smoking patterns after conception,[5 10-15] and although it is logical to try to minimise fetal exposure to tobacco smoke by offering cessation support in early pregnancy, support may be welcomed at other times in gestation. Three prospective, longitudinal studies have reported smoking patterns in pregnancy;[10 11 15] however, two are over 20 years old[10 15] (including the only one to have been conducted in the UK[10]), the third had a smoker response rate of only 25%[11] and none of these studies report when in pregnancy women have tried to quit.[10 11 15] To help focus smoking cessation interventions at the most effective leverage points, we need contemporary, longitudinal data on the smoking and quitting behaviours of pregnant women and also on their receptivity to cessation support. Consequently, we investigate the frequency of pregnant smokers' quit attempts and the factors associated with these. We also attempt to quantify individual-level changes in smoking behaviour during these times.

For peer review only

METHODS

Study design and configuration

A longitudinal cohort study was undertaken; eligible women were aged 16 or over, 8 to 26 weeks pregnant and currently smoking or had smoked regularly during the 3 months immediately prior to pregnancy. Surveys were conducted at recruitment (8-26 weeks gestation), in late pregnancy (34-36 weeks gestation) and 3 months after delivery. Full methods and characteristics of the recruited participants, including factors associated with being a current smoker, are detailed elsewhere.[16] STROBE guidance was used for reporting.[17] Ethical approval was given by Derbyshire Research Ethics Proportionate Review Sub-Committee.

Study setting and regimen

Women were recruited between August 2011 and August 2012 when attending routine hospital or ultrasound appointments at two antenatal clinics within Nottingham University Hospitals National Health Service Trust (City Hospital and Queen's Medical Centre). Women attending clinics first completed a screening questionnaire and those eligible and willing completed a baseline questionnaire and were sent follow-up questionnaires by post or via an email web-link.

Measurements

At baseline, 'recent ex-smokers' were women who reported not smoking currently, but had done so during the 3 months before pregnancy. On later questionnaires, women who reported quitting smoking since the previous survey were also defined as 'recent ex-smokers'. At any time, if women reported smoking at least occasionally, they were

For peer review only

classified as smokers and were asked about this and any quit attempts (including whether attempts had lasted at least 24 hours). On all questionnaires women were asked about future intentions to quit, urges to smoke and the 'Heaviness of Smoking index' (HSI) was calculated as the sum of scores from two items of the Fagerström Test of Cigarette Dependence[18] (scores range, 0 to 6; higher score indicates greater cigarette dependence). Women were also asked to rate their interest in receiving different forms of cessation support on a Likert scale ranging from 1 'not at all' to 5 'extremely'.

Statistical analysis

In order to quantify the proportion of quit attempts made after the first trimester of pregnancy, we aimed to recruit 850 participants.[16] Analyses were conducted using Stata version 14.0 (Stata Corp, College Station, TX, USA).

Descriptive statistics summarised participants' characteristics and smoking behaviour at each time point and we compared those responding to all three questionnaires with those who did not using chi-squared and t-tests for categorical and continuous variables respectively, with p values of <0.05 deemed significant. Characteristics found to be significantly associated with non-completion of later questionnaires, and hence absence of smoking data, were used with multiple imputation to adjust for attrition of smoking behaviour at later time points.

An exploratory analysis was performed to investigate the factors associated with reporting having made a quit attempt of any duration on the baseline questionnaire. For this analysis items were dichotomised; six self-efficacy items that had high internal consistency (Cronbach's α=0.95)[19] were combined into a single score out of 30 with women scoring ≥ 25 considered to have high self-efficacy. Univariable logistic regression

For peer review only

analysis was used to calculate an OR with 95% CIs for each variable (age,[20 21] ethnicity,[22] qualifications held,[20] previous pregnancy,[20 21 23 24] number of cigarettes smoked per day, HSI,[21 23 25] partner smoking status,[21] occupation,[26] planned or surprise pregnancy,[20 27-29] depression, long term disability or mental illness,[30] smoking beliefs and self-efficacy[31]). Variables which showed a significant association ($p<0.05$) in the univariable analysis were included in a multivariable logistic regression model. Variables that achieved significance ($p<0.05$) remained in the multivariable model and all non-significant variables identified from the univariable analysis were re-entered into the model consecutively to assess whether they became significant. The final multivariable model included only significant variables ($p<0.05$). A likelihood ratio test identified that age should be included in the multivariable analysis as a continuous variable. Where collinearity between variables was anticipated (for example, the number of cigarettes smoked per day and HSI), we included the variable that resulted in a better fitting model. As this analysis only included baseline data, we did not need to take account of attrition.

Multiple imputation for missing outcome data for smoking in late pregnancy (34-36 weeks gestation) and at 3 months after delivery was performed using Stata's mi command, based on 20 iterations. The outcomes were imputed using multivariable logistic regression models based on the following baseline variables: age, smoking status, gestation, general health, depression, previous pregnancy, smoking in previous pregnancy, smoking urges, qualifications, and ethnicity. All baseline variables were included in the analysis in dichotomised format. The percentage of women smoking at each outcome was obtained using Rubin's rule.[32] Where necessary, an augmented regression approach was used to overcome issues relating to perfect prediction during the multiple imputation.

For peer review only

RESULTS

Figure 1 summarises questionnaire response rates. Of 1101 eligible women, 966 (88%) completed the baseline survey, and 850 (77%) consented to receive the later surveys. Questionnaires were returned by 509 (59.9%) in late pregnancy, and 476 (56.0%) at 3 months postpartum with 407 (47.8%) women completing all three.

[**Figure 1** Diagram showing recruitment and flow of participants through the study]

Participants had similar socio-demographic characteristics to those in previous pregnancy cohorts and have been reported elsewhere.[16] Just over half (488, 57.4%) were current smokers and 729 (85.7%) of the 850 women in the cohort reported their longer-term quitting intentions (data missing for 121 (14.2%)). Of these 729 women, 424 (58.2%) planned to stop smoking permanently, 21 (2.9%) intended to stop until their baby was born, and 181 (24.8%) were unsure; however, 103 (14.1%) did not plan to stop. Amongst the 272 smokers who reported a quit attempt at baseline, 14 (7.6%) reported stopping for >30 days, 32 (12.2%) for 7-30 days, 126 (48.1%) for 1-6 days, and 84 (32.1%) for <24 hours.

Responding to all three surveys was associated with being older, less cigarette dependant, primiparous, in a planned pregnancy and being a 'recent ex-smoker' at the outset of the study (Table 1).

For peer review only

Table 1 Comparison of participants who completed all three questionnaires with those who completed either one or two questionnaires.

**Characteristic**	**Completed all follow-up surveys N = 397 (46.7%)**	**Did not complete all surveys N = 453 (53.3%)**	**P value**
	**N (%)**	**N (%)**	
**Weeks gestation** (mean, SD)	15.8 (4.1)	15.4 (4.1)	0.14
**Age, years** (mean, SD)	26.5 (5.6)	25.2 (5.5)	<0.001**
**Baseline smoking status**			
Current smoker	199 (50.1)	289 (63.8)	
Ex-smoker	198 (49.9)	164 (36.2)	<0.001**
**Previous pregnancy**			
Not been pregnant before	143 (36.0)	132 (29.1)	
Been pregnant before	250 (63.0)	314 (69.3)	0.037*
**Partner smoking**			
Partner is not a current smoker/no partner	158 (39.8)	187 (41.3)	
Partner is a current smoker	236 (59.4)	263 (58.2)	0.67
**Current smokers only:**			
**Reported quit attempt since learning of pregnancy/previous questionnaire/birth of baby [a]**			
Yes	115 (57.8)	157 (54.3)	
No	78 (39.2)	122 (42.2)	0.47
**Heaviness of Smoking Index**			
Low dependence (0-2)	140 (70.4)	170 (58.8)	
Moderate dependence (3-4)	46 (23.1)	100 (34.6)	
High dependence (5-6)	1 (0.5)	7 (2.4)	0.004*

*significant at <0.05

**significant at <0.001

[a] Quit attempts: at baseline, since finding out about the pregnancy; at follow-up 1, since completing previous survey; follow-up 2, since birth of baby

For peer review only

Figure 2 shows a preliminary descriptive analysis of smoking behaviour across pregnancy within the 397 participants who returned all three questionnaires and illustrates variability in individual's smoking behaviour. Of note, 13.5% (5/37) of women who had stopped smoking in the 3 months before pregnancy were smoking again 3 months after childbirth, whereas 34.2% (55/161) of women who reported that they had quit after finding out they were pregnant had returned to smoking 3 months postpartum. As these data are not adjusted for non-response at follow-up, they may not be consistent with adjusted figures reported below.

[**Figure 2** Change in smoking behaviour between early pregnancy, late pregnancy and postpartum amongst respondents to all 3 questionnaires (N = 397)]

Table 2 shows findings from univariable and multivariable analyses that investigated factors associated with baseline current smokers having reported making a quit attempt earlier in pregnancy. As these analyses only used baseline data, adjustment for attrition was not needed. After the multivariable modelling, four factors were independently associated with reporting previous quit attempts at baseline: smoking fewer daily cigarettes, agreeing that smoking during pregnancy can seriously harm the baby, being primiparous and having a planned pregnancy.

For peer review only

Table 2 Univariable and multivariable associations with previous quit attempts in pregnancy at baseline

			Univariable Model		Multivariable Model	
**Variable**	**Current smokers in each response category N**	**Women who made a quit attempt N (row %)**	**Odds Ratio (95% confidence intervals)**	**P value**	**Odds Ratio (95% confidence intervals)**	**P value**
**Age, years**						
<20	97	64 (66.0)	1.00			
21-25	179	101 (56.4)	0.63 (0.37-1.07)			
26-30	123	57 (46.3)	0.42 (0.24-0.74)	0.026		
Over 31	86	48 (55.8)	0.64 (0.35-1.19)			
**Age, years Mean (SD)**	25.3(5.4)	24.9 (5.7)	0.97 (0.94-1.01)	0.11		
**General Health**						
Excellent	68	45 (66.2)	1.00			
Good	348	178 (51.2)	0.49 (0.28-0.87)			
Fair	68	47 (69.1)	0.95 (0.46-1.97)	0.016		
Poor	2	1 (50.0)	0.44 (0.03-7.5)			
**Qualifications held**						
None	121	50 (41.3)	1.00			
GCSEs or above	367	222 (60.5)	2.27 (1.49-3.46)	0.0001		
**Previous pregnancy**						
Yes	346	169 (48.8)	1.00		1.00	
No	137	101 (73.7)	3.17 (2.02-4.98)	<0.0001	2.20 (1.33-3.66)	0.0019
**Number of cigarettes smoked per day**						
≤5	191	136 (71.2)	1.00		1.00	
6-10	151	86 (57.0)	0.56 (0.36-0.88)	<0.0001	0.65 (0.39-1.07)	
≥11	131	45 (34.4)	0.22 (0.14-0.36)		0.28 (0.16-0.48)	<0.0001
**HSI**						
Low dependence	310	196 (63.2)	1.00			
Moderate dependence	146	61 (41.8)	0.43 (0.29-0.64)	<0.0001		
High dependence	8	2 (25.0)	0.19 (0.04-0.98)			
**Urge to smoke in last 24 hours**						

For peer review only

No urges	23	14 (60.9)	1.00			
Urges	447	251 (56.2)	0.97 (0.42-2.24)	0.95		
**Strength of urges to smoke in last 24 hours**						
No urges	31	16 (51.6)	1.00			
Weak	334	194 (58.1)	1.28 (0.60-2.70)	0.82		
Strong	103	59 (57.3)	1.26 (0.55-2.86)			
**Partner smoking status**						
Non-smoking partner	111	57 (51.4)	1.00			
Smoking partner	334	189 (56.6)	0.88 (0.57-1.37)	0.50		
No partner	41	26 (63.4)	1.38 (0.69-2.73)			
**Home Ownership**						
Rent/other	427	234 (54.8)	1.00	0.47		
Own home	57	35 (61.4)	1.23 (0.70-2.17)			
**Current employment**						
Not in current paid work	324	163 (50.3)	1.00	0.0005		
In current paid work	164	109 (66.5)	2.01 (1.35-2.99)			
**Usual occupation**						
Manual/not-applicable	351	194 (55.3)	1.00	0.13		
Non-manual	75	46 (61.3)	1.50 (0.88-2.57)			
**Ethnicity**						
White British	447	250 (55.9)	1.00			
Other	39	21 (53.9)	1.05 (0.53-2.10)	0.88		
**Timing of pregnancy**						
Planned	171	110 (64.3)	1.00		1.00	
Surprise	312	158 (50.6)	0.59 (0.40-0.87)	0.007	0.53 (0.34-0.82)	0.0045
**Felt depressed or hopeless in last month**						
Yes	144	88 (61.1)	1.00	0.21		
No	338	181 (53.6)	0.77 (0.52-1.16)			
**Long term disability or**						

For peer review only

**mental illness**						
Yes	66	34 (51.5)	1.00			
No	416	234 (56.3)	1.25 (0.74-2.11)	0.41		
**Smoking during pregnancy can harm your baby**						
Disagree	211	81 (38.4)	1.00		1.00	
Agree	266	183 (68.8)	4.08 (2.76-6.02)	<0.0001	4.23 (2.76-6.48)	<0.0001
**Self-efficacy in quitting**						
Low	412	220 (53.4)	1.00			
High	47	35 (74.5)	3.72 (1.68-8.21)	0.001		

For peer review only

At baseline, smokers reported making a median (IQR) of 2 (1-3) quit attempts lasting at least 24 hours since discovering they were pregnant; in later pregnancy 2 (1-5) quit attempts were reported since completing the first questionnaire and in the postpartum 2 (1-4) quit attempts were reported since childbirth (unadjusted data). The median number of quit attempts made by those who smoked across their pregnancy (smokers who completed both baseline and late pregnancy questionnaires, n=177) was 3 (IQR 1-6); these data were highly skewed with a range of 0-60 24-hour quit attempts reported.

Table 3 shows data on smoking rates, quitting behaviour and quit intentions at the three time points adjusted for non-response, as appropriate, using multiple imputation; raw (unadjusted) data are included for reference in a supplementary online table (Appendix Table S1). Adjusted figures show no evidence that smoking rates changed in pregnancy; the proportion of smokers was 57.4% (95% CI 54.1-60.7) at baseline and 59.1% (95% CI 54.9-63.4) in late pregnancy. However, by 3 months postnatally, the adjusted proportion of current smokers was 67.1% (95% CI 62.7-71.5). Over half (55.7%, 95% CI 51.3-60.1) of smokers reported making quit attempts since becoming pregnant and 51.3% (95% CI 44.7- 58.0) did so between early and late pregnancy; however, only 27.4% (95% CI 21.7-33.2) reported trying to stop after childbirth. The proportion of women who intended to try quitting within the next month fell as pregnancy progressed from 40.4% (95% CI 36.1-44.8) at baseline to 29.7% (95% CI 23.8-35.6) in late pregnancy and only 14.2% (95% CI 10.0-18.3) postpartum.

For peer review only

Table 3 Smoking behaviours reported in pregnancy and the postpartum adjusted for non-response at late pregnancy and 3 months postpartum using multiple imputation

**Characteristic**	**Baseline (Early pregnancy)**			**Late pregnancy**		**Postpartum**	
	**n**	**%**	**95% CI**	**%**	**95% CI**	**%**	**95% CI**
**Current smokers**	488	57.4	54.1-60.7	59.1	54.9-63.4	67.1	62.7-71.5
**Reported quit attempt since learning of pregnancy/previous questionnaire/birth of baby [a]**							
Yes	272	55.7	51.3-60.1	51.3	44.7-58.0	27.4	21.7-33.2
No	200	41.0	36.7-45.4	48.7	42.0-55.3	72.6	66.8-78.3
**If have made a quit attempt, attempt lasted at least 24 hours**							
Yes	178	65.4	59.6-71.0	78.9	71.6-86.2	67.8	55.8-79.8
No	90	33.1	27.7-38.9	21.1	13.8-28.4	32.2	20.2-44.2
**Cigarettes per day**							
0-10	342	70.1	65.9-74.0	68.3	63.2-73.3	60.0	54.3-65.7
≥11	131	26.8	23.1-31.0	31.6	26.6-36.7	40.0	34.3-45.7
**How soon after waking smoke first cigarette**							
≤30 minutes	260	53.3	48.8-57.7	48.6	43.1-54.2	48.2	41.8-54.6
≥31 minutes	206	42.2	37.9-46.7	51.4	45.8-56.9	51.8	45.4-58.2
**Heaviness of Smoking Index**							
Low dependence (0-2)	310	63.5	59.1-67.7	68.1	62.5-73.8	65.7	59.1-72.3
Moderate-high dependence (3-6)	154	32.6	27.6-35.8	31.9	26.2-37.5	34.3	27.7-40.9
**Intention to quit smoking**							
Intending to quit within next 30 days	197	40.4	36.1-44.8	29.7	23.8-35.6	14.2	10.0-18.3
Intending to quit within next 3 months/not seriously planning to quit	252	51.6	47.2-56.1	70.3	64.4-76.2	85.8	81.7-90.0
**Urges to smoke**							
**How often felt urges to smoke in previous 24 hours**							
No/few urges/don't know	707	83.2	80.5-85.5	82.0	78.7-85.3	78.7	74.8-82.6
Frequent urges (a lot of the time-all of the time)	135	15.9	13.6-18.5	18.0	14.7-21.3	21.3	17.4-25.2
**Strength of urges to smoke in previous 24 hours**							
No urges/slight-moderate urges/don't know	738	86.8	84.4-88.9	82.8	79.0-86.5	83.0	79.0-87.0
Strong-extremely strong urges	109	12.8	10.7-15.2	17.2	13.5-21.0	17.0	13.0-21.0

[a] Quit attempts: at baseline, since finding out about the pregnancy; at follow-up 1, since completing previous survey; follow-up 2, since birth of baby

For peer review only

Adjusted data show some differences in the rates of women restarting smoking according to when they report that they initially quit. Among women who had either not smoked in the 3 months before pregnancy or during early pregnancy (before completing the baseline questionnaire), 10.2% (95% CI 6.5-13.9) reported smoking on the late pregnancy questionnaire and 31.2% (95% CI 25.2-37.2) did so at 3 months postpartum. However, if this information is broken down further, for those who said they quit *prior* to becoming pregnant, only 2.5% reported smoking by late pregnancy and 17.8% (95% CI 6.1-29.5) reported smoking 3 months postpartum. Whereas, of those who reported quitting *after* finding out they were pregnant (but before completing the baseline questionnaire), 11.6% (95% CI 7.3-15.9) were smoking by late pregnancy, and 34.4% (95% CI 27.6-41.2) were smoking 3 months postpartum. By comparison, among smokers at baseline who reported not smoking in late pregnancy, 42.9% (95% CI 24.6-61.3) were smoking 3 months after delivery. Overall, of women who reported abstinence on the late pregnancy questionnaire, 26.2% (95% CI 20.3-32.2) had relapsed by 3 months post-delivery.

DISCUSSION

To our knowledge, this is the first UK study to use prospectively collected, longitudinal data to quantify changes in smoking behaviour during pregnancy and postnatally. Despite over 50% of smokers reporting quit attempts across all three trimesters, there was no evidence that overall smoking rates changed between joining the study at around 8-24 weeks gestation and late pregnancy. Within 3 months of giving birth, around one third of women who achieved abstinence before or during early pregnancy had returned to smoking. However, we observed a trend, not previously reported in longitudinal data, whereby those who quit before pregnancy may be less likely to return to smoking postpartum than those that quit on learning of their pregnancy; those that only achieved abstinence in late pregnancy appeared to be most likely to return to smoking

For peer review only

postpartum. Women's motivation to try quitting was lowest in the first 3 months following childbirth; only around a quarter tried quitting during this time and far fewer reported intending to quit in the immediate future than had done so at either pregnancy time point.

A limitation is that, although we followed women longitudinally, at each data collection point we relied on self-reported data and recall of smoking behaviour in the immediate past, so we cannot be completely sure that reports are valid. However, a number of factors should have minimised any misreporting of smoking behaviour: no intervention was tested and there was no expectation that participants should try stopping; researchers emphasised that responses were of interest irrespective of smoking status and, as women completed questionnaires at three stages, they did not have to recollect their behaviour over long periods. Additionally, studies looking at both self-report and biochemically-validated smoking data suggest that self-reported smoking can be both accurate and reliable.[15 33] It is possible that pregnant women who were concerned about the stigma of smoking may have avoided participation; we do not know how this might have affected findings, but women who consented to join the cohort had similar characteristics to those who declined.[16] As the survey was conducted in just two Nottingham hospitals, it is hard to say how far findings can be generalised. To help assess generalisability, we included survey items that permitted comparison with previous studies; we found our participants who continued to smoke in early pregnancy were similar to pregnant smokers enrolled in other major UK cohorts.[16] This suggests that the principal findings may apply to pregnant smokers in the UK generally. Likewise, although absolute smoking rates and smoking cessation advice and treatment may vary, pregnant smokers from other high income countries generally have similar characteristics to those in the UK.[20 34 35] Therefore, it could be considered reasonable to extrapolate many of our findings to pregnant smokers in high income countries generally. Although we had very high rates of eligible women joining the cohort, a

For peer review only

further limitation was that attrition was relatively high, with response rates to the two later questionnaires of 60% and 56%. This is a common problem with longitudinal studies,[36] and as young, pregnant smokers were likely to be a particularly difficult group to maintain contact with, we used a number of recommended methods to try to maximise response rates.[16 36 37] However, rather than simply relying on incomplete data, we have tried to address non-response bias by adjusting later surveys' findings using multiple imputation. In addition, differences in characteristics between the whole cohort and those that responded to all three surveys (Table 1) need to be considered when viewing the unadjusted smoking 'trajectory' analysis shown in Figure 2.

The originality of this study is a key strength. As previously mentioned, we could only find three previous observational studies in which pregnant women's smoking behaviours were longitudinally recorded at more than one time point in pregnancy and these have limitations.[10 11 15] All other studies investigating individuals' changes in smoking behaviour in pregnancy have asked about this retrospectively after pregnancy or at only one time point, and in contrast, we collected longitudinal data during and after pregnancy. We particularly focussed on quit attempts and quitting intentions, rather than purely on smoking status at different time points, and are not aware of any other longitudinal studies that have attempted this. Additionally, for the first time we have reported 'attrition-adjusted' rates of smoking or quitting in later pregnancy and postpartum. If we had only used cross-sectional data, we might have underestimated the proportion of smokers in later pregnancy and in the postpartum. We believe that we present the most robust available data documenting changes in smoking status and quitting behaviours across pregnancy and into postpartum, for women who are not participating in an intervention study.

The finding that most women in our cohort had quit in the early stages of pregnancy

For peer review only

(before joining the study), and that smoking rates did not change between the second trimester and 36 weeks' gestation is consistent with cross-sectional estimates for smoking prevalence obtained in a large US study, which reported these by month of pregnancy.[38] In that study, smoking prevalence at 1 month gestation was 26%, then between the fourth and eighth month of gestation, smoking rates each month were 13-14%.[38] Other retrospective studies have found that most women who successfully quit are likely to achieve this soon after finding out they are pregnant,[5 39] often within the first few days.[39] Many quit spontaneously after discovering they are pregnant.[40] Therefore, it seems that after the early stages of pregnancy, despite still reporting quit attempts, women's smoking behaviour actually undergoes very little change.

One study found that 70% of pregnant women making their first quit attempt did so in their first trimester; however, these data were collected up to 5 years after delivery, and only considered first quit attempts.[14] We found that some women made multiple quit attempts throughout pregnancy and most reported cutting down since becoming pregnant. Far fewer women reported making quit attempts in the 3 months after childbirth than they did during pregnancy. Even in early pregnancy, around half of women had no intention to quit within the next 30 days; intention to quit in the short term was even lower in late pregnancy, and was lowest of all postpartum. This diminishing intention to quit has not been reported before and could be considered when designing and delivering cessation interventions.

We found that women who were primiparous, smoked fewer cigarettes per day, had a planned pregnancy and believed smoking during pregnancy could seriously harm their baby were more likely to have made a quit attempt during early pregnancy. These findings are comparable to previous literature examining the characteristics of pregnant smokers who successfully achieve cessation. Primiparous women have previously been

For peer review only

found to be more likely to successfully quit smoking.[20 21 23 24] This may be because women who have smoked throughout a previous pregnancy without experiencing complications may view the risks of smoking during pregnancy differently to primiparous women, and therefore be less motivated to make a quit attempt.[21] Similarly, previous studies have found that heavier smoking is negatively associated with successfully quitting in pregnancy,[21 23 25] and heavier smokers are less likely to have high motivation to quit during pregnancy.[25] Women whose pregnancies are unintended have previously been found to be more likely to continue smoking during pregnancy,[20 27-29] and likewise pregnant smokers who do not report concern about the effect smoking might have on the health of their unborn baby were more likely to have low motivation to quit smoking.[25] These findings identify women who are most likely to make a quit attempt and will potentially benefit the most from NHS support. Heavier smokers and women in second or later and unplanned pregnancies who are less likely to try quitting may require different, more intensive or tailored forms of support.

Implications for practice

Although our data suggest that motivation to quit may be strongest in early pregnancy, some women will be receptive to quitting at any time, as indicated by their multiple quit attempts throughout pregnancy, and this confirms that it is important to discuss smoking with women at every appointment and to refer them for stop smoking support.[9] Preventing resumption of smoking after pregnancy is a critical public health issue; if women restart their lifelong health is at risk, and their infants are more likely to be exposed to second-hand smoke[41] and to eventually become smokers.[42] Women often need help to resist returning to smoking after childbirth, but there are currently few effective interventions for this.[43] Women appear to be more inclined to consider quitting during pregnancy than in postpartum, and this is important when designing interventions. However, some postpartum women do make quit attempts, or may be

For peer review only

planning to quit in the medium term, so engaging with them again after birth, to think about planning for this in the medium term, rather than immediately, might be a successful option. Previous studies have shown that women who quit spontaneously early in pregnancy are likely to be different and more successful than those who quit later,[40] and we found that women appear to be more likely to return to smoking after childbirth the later in pregnancy they quit. Therefore, exploring potential reasons for this, for example demographic factors or women's intentions, could help to identify if different women may benefit from alternative approaches to help prevent relapse, perhaps by developing more tailored interventions. Although quit attempts might suggest receptivity to quitting, what is not well understood is how interest in smoking cessation support may change during pregnancy.

Conclusions

Many pregnant women who smoke attempt quitting throughout their pregnancy, but this makes little difference to overall smoking rates. After giving birth, most smokers seem less inclined to make further quit attempts and many who quit in early pregnancy return to smoking. Women who quit in late pregnancy may be most likely to return to smoking after childbirth, whilst those who stopped prior to pregnancy may be least likely to relapse. It therefore imperative to discuss smoking with women, including recent ex-smokers, throughout pregnancy and postpartum, and to continue to offer and provide specialist stop smoking support.

For peer review only

ACKNOWLEDGEMENTS

The authors would like to thank all participants and staff involved in this study, and Nottingham University Hospitals NHS Trust for facilitating this research. We also thank Rachel Whitemore for her invaluable help with this study.

CONTRIBUTORS

SC helped conceive the study, made a substantial contribution to the development of the protocol and questionnaires, assisted with day-to-day troubleshooting during the data-collection phase, and drafted and revised this manuscript. SO helped design the data collection process, recruited participants into the cohort, managed the day-to-day running, assisted with data analysis, and contributed to the drafting of this manuscript. JL-B contributed to the development of the study protocol and questionnaires, advised on analysis, and contributed to the preparation of this manuscript. EB undertook the analyses and interpretation of data in Table 2 as part of a BMedSci project and contributed to the preparation of this manuscript. LV assisted with data analysis and interpretation, and contributed to the preparation of this manuscript. KB helped design the data collection process, recruited participants into the cohort, managed the day-to-day running, and contributed to the preparation of this manuscript. FN, MU, KEP and SS all contributed to the development of the study protocol and questionnaires, contributing expertise from their own particular knowledge base, and to the preparation of this manuscript. TC conceived the study, and made substantial contributions to the development of the study protocol and questionnaires, and to the preparation of this manuscript. All authors read and approved the final manuscript. Rachel Whitemore assisted with study administration, telephone follow-ups, and data entry.

For peer review only

FUNDING

This article presents independent research funded by the National Institute for Health Research (NIHR) under its Programme Grants for Applied Research Programme (Grant Number RP-PG 0109-10020). The views expressed in this paper are those of the authors and not necessarily those of the NHS, the NIHR or the Department of Health.

SC, SO, JLB, KB, FN, MU, SS and TC are members of the UK Centre for Tobacco and Alcohol Studies (UKCTAS), a UKCRC Public Health Research Centre of Excellence. UKCTAS receives core funding from the British Heart Foundation, Cancer Research UK, Economic and Social Research Council, Medical Research Council and the Department of Health under the auspices of the UK Clinical Research Collaboration. SC, SO, KB, SS and TC are members of the NIHR School for Primary Care Research. TC acknowledges the support of the National Institute for Health Research (NIHR) Collaboration for Leadership in Applied Health Research.

COMPETING INTERESTS

KP is a trustee of The Equality Trust (a registered charity), and receives occasional honoraria, all of which are donated to The Equality Trust or for student support at the University of York. In the last 5 years TC has been paid honoraria on 2 occasions for speaking at meetings or conferences organised by Pierre Fabre Laboratories (a nicotine replacement therapy manufacturer).

Other authors have no conflicts of interest to report.

ETHICS APPROVAL

Derbyshire Research Ethics Proportionate Review Sub-Committee gave ethical approval.

For peer review only

PROVENANCE AND PEER REVIEW

Not commissioned; externally peer reviewed.

DATA SHARING STATEMENT

The dataset is still subject to further analyses. Relevant anonymised data available from the authors on reasonable request.

For peer review only

REFERENCES

1. British Medical Association. Smoking and reproductive life: The impact of smoking on sexual, reproductive and child health. London: BMA, 2004.

2. Cnattingius S. The epidemiology of smoking during pregnancy: smoking prevalence, maternal characteristics, and pregnancy outcomes. *Nicotine Tob Res* 2004;6(S2):S125-S40. doi: 10.1080/14622200410001669187

3. Jensen KE, Jensen A, Nohr B, et al. Do pregnant women still smoke? A study of smoking patterns among 261,029 primiparous women in Denmark 1997-2005. *Acta Obstet Gynecol Scand* 2008;87(7):760-67. doi: Doi 10.1080/00016340802179814

4. Mohsin M, Bauman AE, Forero R. Socioeconomic correlates and trends in smoking in pregnancy in New South Wales, Australia. *J Epidemiol Community Health* 2011;65(8):727-32. doi: 10.1136/jech.2009.104232 [published Online First: 2010/09/16]

5. McAndrew F, Thompson J, Fellows L, et al. Infant Feeding Survey 2010: Health and Social Care Information Centre, 2012.

6. Tong VT, Dietz PM, Morrow B, et al. Trends in smoking before, during, and after pregnancy--Pregnancy Risk Assessment Monitoring System, United States, 40 sites, 2000-2010. *MMWR Surveill Summ* 2013;62(6):1-19. [published Online First: 2013/12/19]

7. Bloch M, Althabe F, Onyamboko M, et al. Tobacco use and secondhand smoke exposure during pregnancy: An investigative survey of women in 9 developing nations. *Am J Public Health* 2008;98(10):1833-40. doi: Doi 10.2105/Ajph.2007.117887

8. Fahy SJ, Cooper S, Coleman T, et al. Provision of smoking cessation support for pregnant women in England: results from an online survey of NHS stop smoking services for pregnant women. *BMC Health Serv Res* 2014;14(1):107. doi: 10.1186/1472-6963-14-107

9. National Institute for Health and Clinical Excellence. Public health guidance 26: How to stop smoking in pregnancy and following childbirth. London, 2010.

10. Munafo MR, Heron J, Araya R. Smoking patterns during pregnancy and postnatal period and depressive symptoms. *Nicotine Tob Res* 2008;10(11):1609-20.

11. Eiden RD, Homish GG, Colder CR, et al. Changes in Smoking Patterns During Pregnancy. *Subst Use Misuse* 2013;48(7):513-22. doi: doi:10.3109/10826084.2013.787091

12. Mumford EA, Hair EC, Yu T-C, et al. Women's Longitudinal Smoking Patterns from Preconception Through Child's Kindergarten Entry: Profiles of Biological Mothers of a 2001 US Birth Cohort. *Matern Child Health J* 2014;18(4):810-20. doi: 10.1007/s10995-013-1305-y

For peer review only

13. Fitzpatrick KE, Gray R, Quigley MA. Women's Longitudinal Patterns of Smoking during the Pre-Conception, Pregnancy and Postnatal Period: Evidence from the UK Infant Feeding Survey. *PLoS One* 2016;11(4):e0153447. doi: 10.1371/journal.pone.0153447

14. Pickett KE, Wakschlag LS, Dai L, et al. Fluctuations of maternal smoking during pregnancy. *Obstet Gynecol* 2003;101(1):140-47.

15. Pickett KE, Rathouz PJ, Kasza K, et al. Self-reported smoking, cotinine levels, and patterns of smoking in pregnancy. *Paediatr Perinat Epidemiol* 2005;19(5):368-76.

16. Orton S, Bowker K, Cooper S, et al. Longitudinal cohort survey of women's smoking behaviour and attitudes in pregnancy: study methods and baseline data. *BMJ Open* 2014;4(5):e004915. doi: 10.1136/bmjopen-2014-004915

17. von Elm E, Altman DG, Egger M, et al. The Strengthening the Reporting of Observational Studies in Epidemiology (STROBE) statement: guidelines for reporting observational studies. *Lancet* 2007;370(9596):1453-57. doi: 10.1016/s0140-6736(07)61602-x

18. Heatherton TF, Kozlowski LT, Frecker RC, et al. Measuring the heaviness of smoking: using self-reported time to the first cigarette of the day and number of cigarettes smoked per day. *Br J Addict* 1989;84(7):791-99.

19. Cronbach LJ. Coefficient alpha and the internal structure of tests. *Psychometrika* 1951;16(3):297-334. doi: 10.1007/bf02310555

20. Smedberg J, Lupattelli A, Mårdby AC, et al. Characteristics of women who continue smoking during pregnancy: A cross-sectional study of pregnant women and new mothers in 15 European countries. *BMC Pregnancy Childbirth* 2014;14:213. doi: 10.1186/1471-2393-14-213

21. Giglia RC, Binns CW, Alfonso HS. Which women stop smoking during pregnancy and the effect on breastfeeding duration. *BMC Public Health* 2006;6:195. doi: 10.1186/1471-2458-6-195

22. Tong VJ, JR.; Dietz, PM.; D'Angelo, D.; Bombard, JM. Trends in Smoking Before, During, and After Pregnancy — Pregnancy Risk Assessment Monitoring System (PRAMS), United States, 31 Sites, 2000–2005. *MMWR Surveill Summ* 2009;58(SS-4):1-36.

23. Hakansson A, Lendahls L, Petersson C. Which women stop smoking? A population-based study of 403 pregnant smokers. *Acta Obstet Gynecol Scand* 1999;78(3):217-24.

24. Lindqvist R, Aberg H. Who stops smoking during pregnancy? *Acta Obstet Gynecol Scand* 2001;80(2):137-41.

25. Ershoff DH, Solomon LJ, Dolan-Mullen P. Predictors of intentions to stop smoking early in prenatal care. *Tob Control* 2000;9 Suppl 3:III41-5.

For peer review only

26. Graham H, Hawkins SS, Law C. Lifecourse influences on women's smoking before, during and after pregnancy. *Soc Sci Med* 2010;70(4):582-87. doi: http://dx.doi.org/10.1016/j.socscimed.2009.10.041

27. Dott M, Rasmussen SA, Hogue CJ, et al. Association Between Pregnancy Intention and Reproductive-health Related Behaviors Before and After Pregnancy Recognition, National Birth Defects Prevention Study, 1997–2002. *Matern Child Health J* 2010;14(3):373-81. doi: 10.1007/s10995-009-0458-1

28. McCrory C, McNally S. The effect of pregnancy intention on maternal prenatal behaviours and parent and child health: results of an irish cohort study. *Paediatr Perinat Epidemiol* 2013;27(2):208-15. doi: 10.1111/ppe.12027

29. Chisolm MS, Cheng D, Terplan M. The relationship between pregnancy intention and change in perinatal cigarette smoking: an analysis of PRAMS data. *J Subst Abuse Treat* 2014;46(2):189-93. doi: 10.1016/j.jsat.2013.07.010

30. McCormick MC, Brooks-Gunn J, Shorter T, et al. Factors associated with smoking in low-income pregnant women: relationship to birth weight, stressful life events, social support, health behaviors and mental distress. *J Clin Epidemiol* 1990;43(5):441-48.

31. Devries H, Backbier E. Self-efficacy as an important determinant of quitting among pregnant women who smoke: the Ø-pattern. *Prev Med* 1994;23(2):167-74.

32. Rubin DB. Multiple imputation for nonresponse in surveys. New York: John Wiley and Sons 1987.

33. George L, Granath F, Johansson ALV, et al. Self-reported nicotine exposure and plasma levels of cotinine in early and late pregnancy. *Acta Obstet Gynecol Scand* 2006;85(11):1331-37.

34. Lu Y, Tong S, Oldenburg B. Determinants of smoking and cessation during and after pregnancy. *Health Promot Int* 2001;16(4):355-65. doi: 10.1093/heapro/16.4.355

35. Schneider S, Schütz J. Who smokes during pregnancy? A systematic literature review of population-based surveys conducted in developed countries between 1997 and 2006. *Eur J Contracept Reprod Health Care* 2008;13(2):138-47. doi: 10.1080/13625180802027993

36. Ribisl KM, Walton MA, Mowbray CT, et al. Minimizing participant attrition in panel studies through the use of effective retention and tracking strategies: Review and recommendations. *Eval Program Plann* 1996;19(1):1-25. doi: http://dx.doi.org/10.1016/0149-7189(95)00037-2

37. Edwards P, Roberts I, Clarke M, et al. Methods to increase response rates to postal questionnaires. *Cochrane Database Syst Rev* 2007(2):MR000008.

For peer review only

38. Alshaarawy O, Anthony JC. Month-Wise Estimates of Tobacco Smoking During Pregnancy for the United States, 2002–2009. *Matern Child Health J* 2015;19(5):1010-15. doi: 10.1007/s10995-014-1599-4

39. Heil SH, Herrmann ES, Badger GJ, et al. Examining the timing of changes in cigarette smoking upon learning of pregnancy. *Prev Med* 2014;68:58-61. doi: http://dx.doi.org/10.1016/j.ypmed.2014.06.034

40. Solomon LJ, Quinn VP. Spontaneous quitting: Self-initiated smoking cessation in early pregnancy. *Nicotine Tob Res* 2004;6(SUPPL. 2):S203-S16. doi: 10.1080/14622200410001669132

41. Orton S, Jones LL, Cooper S, et al. Predictors of Children's Secondhand Smoke Exposure at Home: A Systematic Review and Narrative Synthesis of the Evidence. *PLoS One* 2014;9(11):e112690. doi: 10.1371/journal.pone.0112690

42. Leonardi-Bee J, Jere ML, Britton J. Exposure to parental and sibling smoking and the risk of smoking uptake in childhood and adolescence: a systematic review and meta-analysis. *Thorax* 2011;66(10):847-55. doi: 10.1136/thx.2010.153379

43. Hajek P, Stead Lindsay F, West R, et al. Relapse prevention interventions for smoking cessation. *Cochrane Database Syst Rev* 2013(8):CD003999. doi: 10.1002/14651858.CD003999.pub4

For peer review only

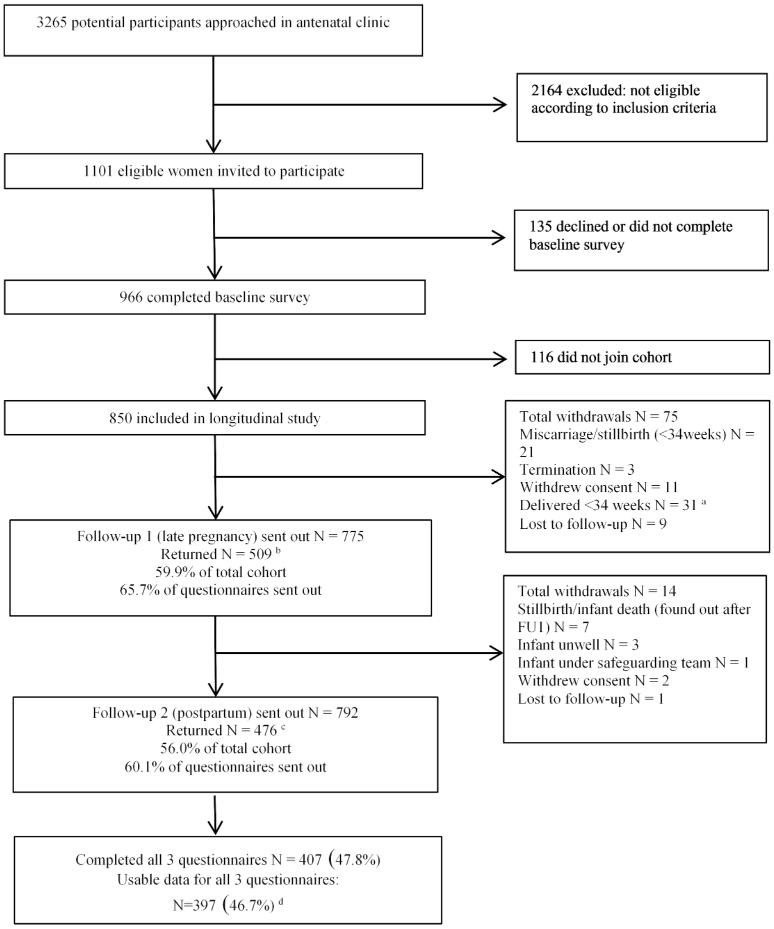

[a] 28 of the participants who delivered before 34 weeks gestation were sent the postpartum questionnaire

[b] 13 excluded who completed a follow-up 1 questionnaire, but weren't eligible (i.e. any women who was not still pregnant at 34 weeks)

[c] 70 participants who did not complete a late pregnancy questionnaire returned the postpartum questionnaire; of these, seven were women who delivered before 34 weeks gestation

[d] 10 excluded who completed all 3 questionnaires, but weren't eligible to complete follow-up 1 questionnaire (i.e. if miscarriage/stillbirth before 34 weeks gestation, terminated pregnancy or gave birth before 34 weeks)

Figure 1 Diagram showing recruitment and flow of participants through the study

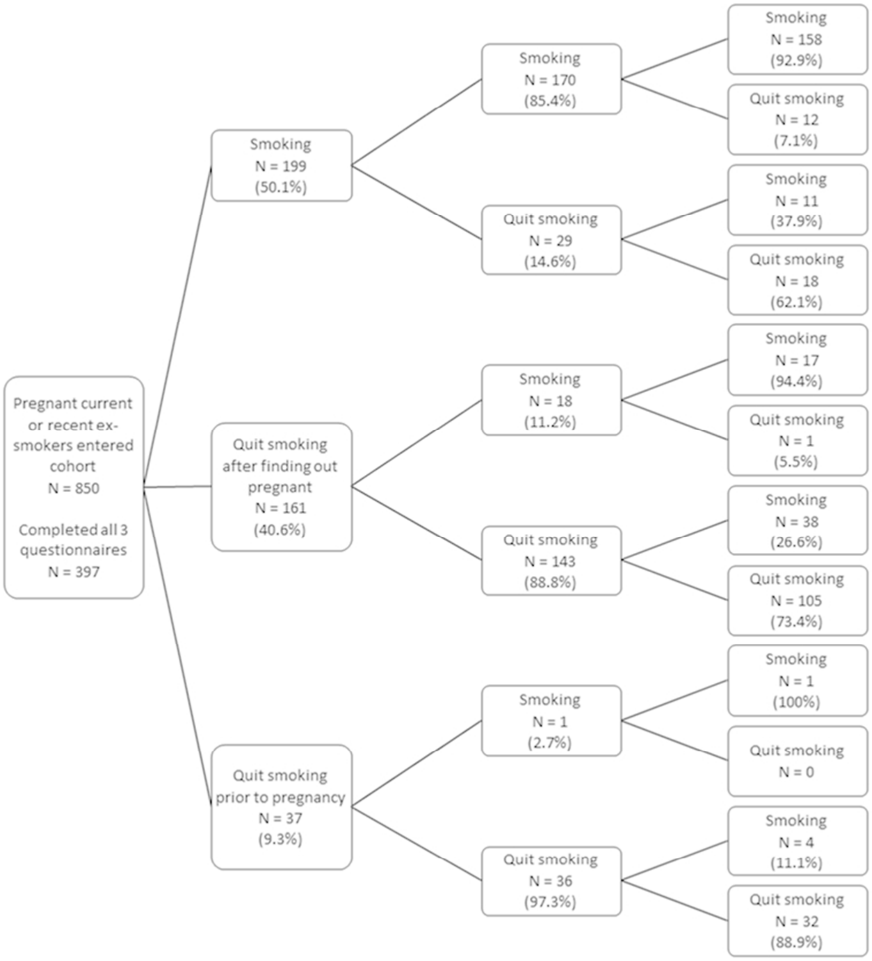

Figure 2 Change in smoking behaviour between early pregnancy, late pregnancy and postpartum amongst respondents to all 3 questionnaires (N = 397)

For peer review only - http://bmjopen.bmj.com/site/about/guidelines.xhtml

Appendix Table S1 Cross-sectional data: participants' smoking behaviour for returned questionnaires only at each follow up point

**Characteristic**	**Baseline (Early pregnancy) N (%)**	**Late pregnancy N (%)**	**Postpartum N (%)**
**Respondents** (response rate)	850	509 (59.9)	476 (56.0)
**Smoking status**			
Current smoker	488 (57.4)	252 (49.5)	281 (59.0)
Recent ex-smoker	362 (42.6)	257 (50.5)	195 (41.0)
**Current smoking behaviour (all participants)**			
Stopped smoking during 3 months prior to pregnancy	61 (7.2)		
Stopped smoking after learning of pregnancy/don't smoke at all now	301 (35.4)	257 (50.5)	195 (41.0)
Smoke occasionally, but not every day now pregnant	117 (13.8)	61 (12.0)	84 (17.6)
Smoke every day, but have cut down since: learning of pregnancy/during pregnancy	304 (35.8)	144 (28.3)	56 (11.8)
Smoke every day, about the same as before/during pregnancy	64 (7.5)	42 (8.3)	80 (16.8)
Smoke every day, tend to smoke more than before/during pregnancy	3 (0.4)	4 (0.8)	59 (12.4)
Missing	0	1 (0.2)	2 (0.4)
**Current smokers only:**			
**Reported quit attempt since learning of pregnancy/previous questionnaire/birth of baby** [a]			
Yes	272 (55.7)	130 (51.6)	78 (27.8)
No	200 (41.0)	119 (47.2)	191 (68.0)
Missing	16 (3.3)	3 (1.2)	12 (4.3)
**If have made a quit attempt, attempt lasted at least 24 hours**			
Yes<br>(% of all current smokers)<br>(% of current smokers making a quit attempt)	178<br>(36.5%)<br>(65.4%)	103<br>(40.9%)<br>(79.2%)	61<br>(21.7%)<br>(78.2%)
No<br>(% of all current smokers)<br>(% of current smokers making a quit attempt)	90<br>(18.4%)<br>(33.1%)	19<br>(7.5%)<br>(14.6%)	6<br>(2.1%)<br>(7.7%)
**Number of quit attempts >24 hours, median (IQR)**	2 (1-3)	2 (1-5)	2 (1-4)
**Cigarettes per day**			
0-5	191 (39.1)	99 (39.3)	102 (36.3)
6-10	151 (30.9)	85 (33.7)	83 (29.5)
11-15	74 (15.2)	32 (12.7)	55 (19.6)
16-20	47 (9.6)	24 (9.5)	30 (10.7)
21-30	8 (1.6)	9 (3.6)	5 (1.8)
≥31	2 (0.4)	0 (0)	1 (0.4)
Missing	15 (3.1)	3 (1.2)	5 (1.8)
**Time to first cigarette**			
< 5 minutes	97 (19.9)	31 (12.3)	28 (10.0)
6-30 minutes	163 (33.4)	79 (31.3)	83 (29.5)
31-60 minutes	89 (18.2)	44 (17.5)	50 (17.8)

For peer review only

>60 minutes	117 (24.0)	91 (36.1)	105 (37.4)
Missing	22 (4.5)	7 (2.8)	15 (5.3)
**Heaviness of Smoking Index (HSI)**			
Low dependence (0-2)	310 (63.5)	178 (70.6)	189 (67.3)
Moderate dependence (3-4)	146 (29.9)	60 (23.8)	75 (26.7)
High dependence (5-6)	8 (1.6)	6 (2.4)	2 (0.7)
Missing	24 (4.9)	8 (3.2)	15 (5.3)
**Intention to quit smoking**			
Intending to quit within next 2 weeks	138 (28.3)	51 (20.2)	20 (7.1)
Intending to quit within next 30 days	59 (12.1)	23 (9.1)	22 (7.8)
Intending to quit within next 3 months	76 (15.6)	73 (29.0)	100 (35.6)
Not seriously intending to quit	176 (36.1)	90 (35.7)	123 (43.8)
Missing	39 (8.0)	15 (6.0)	16 (5.7)

[a] Quit attempts: at baseline, since finding out about the pregnancy; at follow-up 1, since completing previous survey; follow-up 2, since birth of baby

For peer review only

STROBE 2007 (v4) Statement—Checklist of items that should be included in reports of *cohort studies*

Section/Topic	Item #	Recommendation	Reported on page #
**Title and abstract**	1	(*a*) Indicate the study's design with a commonly used term in the title or the abstract	1
		(*b*) Provide in the abstract an informative and balanced summary of what was done and what was found	2
**Introduction**			
Background/rationale	2	Explain the scientific background and rationale for the investigation being reported	5
Objectives	3	State specific objectives, including any prespecified hypotheses	5
**Methods**			
Study design	4	Present key elements of study design early in the paper	6
Setting	5	Describe the setting, locations, and relevant dates, including periods of recruitment, exposure, follow-up, and data collection	6
Participants	6	(*a*) Give the eligibility criteria, and the sources and methods of selection of participants. Describe methods of follow-up	6
		(*b*) For matched studies, give matching criteria and number of exposed and unexposed	
Variables	7	Clearly define all outcomes, exposures, predictors, potential confounders, and effect modifiers. Give diagnostic criteria, if applicable	6-7
Data sources/ measurement	8*	For each variable of interest, give sources of data and details of methods of assessment (measurement). Describe comparability of assessment methods if there is more than one group	6-7
Bias	9	Describe any efforts to address potential sources of bias	7-8
Study size	10	Explain how the study size was arrived at	Previously described in methods paper
Quantitative variables	11	Explain how quantitative variables were handled in the analyses. If applicable, describe which groupings were chosen and why	7-8
Statistical methods	12	(*a*) Describe all statistical methods, including those used to control for confounding	7-8
		(*b*) Describe any methods used to examine subgroups and interactions	n/a
		(*c*) Explain how missing data were addressed	8
		(*d*) If applicable, explain how loss to follow-up was addressed	8
		(*e*) Describe any sensitivity analyses	n/a

For peer review only

**Results**			
Participants	13*	(a) Report numbers of individuals at each stage of study—eg numbers potentially eligible, examined for eligibility, confirmed eligible, included in the study, completing follow-up, and analysed	9 & Figure 1
		(b) Give reasons for non-participation at each stage	Figure 1
		(c) Consider use of a flow diagram	Figure 1
Descriptive data	14*	(a) Give characteristics of study participants (eg demographic, clinical, social) and information on exposures and potential confounders	9, Table 1, Appendix Table S1
		(b) Indicate number of participants with missing data for each variable of interest	Appendix Table S1
		(c) Summarise follow-up time (eg, average and total amount)	n/a
Outcome data	15*	Report numbers of outcome events or summary measures over time	
Main results	16	(*a*) Give unadjusted estimates and, if applicable, confounder-adjusted estimates and their precision (eg, 95% confidence interval). Make clear which confounders were adjusted for and why they were included	Throughout
		(*b*) Report category boundaries when continuous variables were categorized	Throughout
		(*c*) If relevant, consider translating estimates of relative risk into absolute risk for a meaningful time period	n/a
Other analyses	17	Report other analyses done—eg analyses of subgroups and interactions, and sensitivity analyses	
**Discussion**			
Key results	18	Summarise key results with reference to study objectives	17
**Limitations**			
Interpretation	20	Give a cautious overall interpretation of results considering objectives, limitations, multiplicity of analyses, results from similar studies, and other relevant evidence	18-21
Generalisability	21	Discuss the generalisability (external validity) of the study results	18
**Other information**			
Funding	22	Give the source of funding and the role of the funders for the present study and, if applicable, for the original study on which the present article is based	24

*Give information separately for cases and controls in case-control studies and, if applicable, for exposed and unexposed groups in cohort and cross-sectional studies.

Note: An Explanation and Elaboration article discusses each checklist item and gives methodological background and published examples of transparent reporting. The STROBE checklist is best used in conjunction with this article (freely available on the Web sites of PLoS Medicine at http://www.plosmedicine.org/, Annals of Internal Medicine at http://www.annals.org/, and Epidemiology at http://www.epidem.com/). Information on the STROBE Initiative is available at www.strobe-statement.org.

For peer review only

BMJ Open

Smoking and quit attempts during pregnancy and postpartum: a longitudinal UK cohort

Journal:	*BMJ Open*
Manuscript ID	bmjopen-2017-018746.R1
Article Type:	Research
Date Submitted by the Author:	08-Sep-2017
Complete List of Authors:	Cooper, Sue; University of Nottingham, Division of Primary Care<br>Orton, Sophie; University of Nottingham, Division of Primary Care<br>Leonardi-Bee, Jo; Uni Nottingham<br>Brotherton, Emma; University of Nottingham, Division of Primary Care<br>Vanderbloemen, Laura; Imperial College London, Department of Primary Care and Public Health<br>Bowker, Katharine; University of Nottingham, Division of Primary Care<br>Naughton, Felix; University of East Anglia Faculty of Medicine and Health Sciences, School of Health Sciences<br>Ussher, Michael; St George's University of London, Division of Population Health Sciences and Education<br>Pickett, Kate; University of York<br>Sutton, Stephen; University of Cambridge,<br>Coleman, Tim; University of Nottingham, Division of Primary Care
**Primary Subject Heading**:	Smoking and tobacco
Secondary Subject Heading:	Addiction, Epidemiology, Obstetrics and gynaecology, Public health
Keywords:	Smoking cessation, Pregnancy, Longitudinal research, Quit attempts, Postpartum relapse, Survey research

SCHOLARONE™ Manuscripts

Smoking and quit attempts during pregnancy and postpartum: a longitudinal UK cohort

Sue Cooper PhD[1] *, Sophie Orton PhD[1], Jo Leonardi-Bee PhD[2], Emma Brotherton BMedSci[1], Laura Vanderbloemen PhD[3], Katharine Bowker PhD[1], Felix Naughton PhD[4], Michael Ussher PhD[5], Kate E. Pickett PhD[6], Stephen Sutton PhD[7], Tim Coleman MD[1]

[1] Division of Primary Care, University of Nottingham, University Park, Nottingham, NG7 2RD. UK

[2] Division of Epidemiology and Public Health, University of Nottingham, Nottingham, UK

[3] Department of Primary Care and Public Health, Imperial College London, London, UK

[4] School of Health Sciences, University of East Anglia, Norwich, UK

[5] Population Health Research Institute, St Georges, University of London, London, UK

[6] Department of Health Sciences, University of York, York, UK

[7] Behavioural Science Group, University of Cambridge, Cambridge, UK

* Correspondence to: sue.cooper@nottingham.ac.uk

Word Count 3847

For peer review only

ABSTRACT

Objectives

Pregnancy motivates women to try stopping smoking, but little is known about timing of their quit attempts and how quitting intentions change during pregnancy and postpartum. Using longitudinal data, this study aimed to document women's smoking and quitting behaviour throughout pregnancy and after delivery.

Design

Longitudinal cohort survey with questionnaires at baseline (8-26 weeks gestation), late pregnancy (34-36 weeks) and 3 months after delivery.

Setting

Two maternity hospitals in one National Health Service hospital trust, Nottingham, England.

Participants

850 pregnant women, aged 16 or over, who were current smokers or had smoked in the 3 months before pregnancy, were recruited between August 2011 and August 2012.

Outcome measures

Self-reported smoking behaviour, quit attempts and quitting intentions.

Results

Smoking rates, adjusting for non-response at follow-up, were 57.4% (95% CI 54.1-60.7) at baseline, 59.1% (95% CI 54.9-63.4) in late pregnancy and 67.1% (95% CI 62.7-71.5) 3 months postpartum. At baseline, 272 of 488 current smokers had tried to quit since becoming pregnant (55.7%, 95% CI 51.3-60.1); 51.3% (95% CI 44.7-58.0) tried quitting between baseline and late pregnancy and 27.4% (95% CI 21.7-33.2) after childbirth. The percentage who intended to quit within the next month fell as pregnancy

For peer review only

progressed, from 40.4% (95% CI 36.1-44.8) at baseline to 29.7% (95% CI 23.8-35.6) in late pregnancy and 14.2% (95% CI 10.0-18.3) postpartum. Postpartum relapse was lower among women who quit in the 3 months before pregnancy (17.8%, 95% CI 6.1-29.4) than those who stopped between baseline and late pregnancy (42.9%, 95% CI 24.6-61.3)

Conclusions

Many pregnant smokers make quit attempts throughout pregnancy and postpartum, but intention to quit decreases over time; there is no evidence that smoking rates fall during gestation.

Keywords

Smoking cessation, pregnancy, longitudinal research, quit attempts, postpartum relapse, survey research

For peer review only

Strengths and limitations of this study

- As far as we are aware, this is the only study to investigate timing of quit attempts and propensity to stop smoking during pregnancy and postpartum, and to quantify longitudinal changes.
- Smoking behaviour is self-reported rather than validated; misreporting due to recall bias may have been minimised by collecting data at three time points, and by there being no expectation that they should try to stop smoking.
- Later survey findings were adjusted using multiple imputation to help address non-response bias due to attrition.
- As the study was conducted in just one geographical area of the UK and participants were predominantly white British, findings might not be generalisable; however, the demographic profile of participants was similar to that of other UK cohorts of pregnant smokers.

For peer review only

INTRODUCTION

Smoking in pregnancy is associated with increased risks of miscarriage, stillbirth, prematurity, low birth weight, perinatal morbidity and mortality, neo-natal and sudden infant death, infant respiratory problems, poorer infant cognition, and adverse infant behavioural outcomes.[1 2] Internationally, large numbers of pregnant women smoke; with rates between 12% and 22% in high income countries[3-6] and rates increasing in emerging and developing economies.[7] Pregnancy is probably the event which most motivates female smokers to try quitting; for example, in the UK over 50 per cent of pregnant smokers try to stop[5] and pregnant women are, therefore, particularly likely to be interested in receiving cessation support. Some health systems systematically offer such support; in the UK this is largely done in early pregnancy,[8] although official guidance recommends that support is provided throughout gestation.[9]

We are not aware of any studies that have investigated when, in pregnancy, smokers have the greatest propensity to try stopping, the timing of any quit attempts and potential influences on this. Outside of pregnancy and postpartum, most adults tend to have fairly stable smoking behaviour.[10] Although overall smoking rates in pregnancy have declined, a significant proportion of women continue to smoke throughout pregnancy.[11] However, many women who smoke before pregnancy have varied smoking behaviour after conception,[5 11-16] and although it is logical to try to minimise fetal exposure to tobacco smoke by offering cessation support in early pregnancy, support may be welcomed at other times in gestation. In addition, of those that do stop, many relapse within the first few months postpartum.[17 18] Relatively few studies of prenatal smoking behaviour have been longitudinal,[12 13 15 16 19-23] with only two of these following up women postpartum,[12 13] and the only two studies to have been conducted in the UK are now over 20 years old.[12 19] Importantly, none of these studies asked about number of quit attempts or reported when in pregnancy women have tried to quit. To help focus smoking cessation interventions at the most effective leverage points, we need

For peer review only

contemporary, longitudinal data on the smoking and quitting behaviours of pregnant women. Consequently, we investigate the frequency and timing of pregnant smokers' quit attempts, and the factors associated with these. We also attempt to quantify individual-level changes in smoking behaviour during these times.

METHODS

Study design and configuration

A longitudinal cohort study was undertaken; eligible women were aged 16 or over, 8 to 26 weeks pregnant and currently smoking or had smoked regularly during the 3 months immediately prior to finding out they were pregnant. Surveys were conducted at recruitment (8-26 weeks gestation), in late pregnancy (34-36 weeks gestation) and 3 months after delivery. Full methods and characteristics of the recruited participants, including factors associated with being a current smoker, are detailed elsewhere.[24] STROBE guidance was used for reporting.[25] Ethical approval was given by Derbyshire Research Ethics Proportionate Review Sub-Committee.

Study setting and regimen

Women were recruited between August 2011 and August 2012 when attending routine hospital or ultrasound appointments at two antenatal clinics within Nottingham University Hospitals National Health Service Trust (City Hospital and Queen's Medical Centre). Women attending clinics first completed a screening questionnaire and those eligible and willing completed a baseline questionnaire and were sent follow-up questionnaires by post or via an email web-link.

Measurements

For peer review only

At baseline, 'recent ex-smokers' were women who reported not smoking currently, but had done so during the 3 months before finding out they were pregnant. On later questionnaires, women who reported quitting smoking since the previous survey were also defined as 'recent ex-smokers'. At any time, if women reported smoking either every day or occasionally (smoke, but not every day), they were classified as smokers and were asked further details about this, including if they had made any quit attempts and how many of these attempts had lasted at least 24 hours. On all questionnaires women were asked about timescales of future intentions to quit (within next 2 weeks/next 30 days/next 3 months/not planning to quit) (since finding out they were pregnant/since completing the first questionnaire/since the birth of their baby), and about urges to smoke (6 point Likert scale ranging from 'no urges' to 'extremely strong'). The 'Heaviness of Smoking index' (HSI) was calculated as the sum of scores from two items of the Fagerström Test of Cigarette Dependence[26] (scores range, 0 to 6; higher score indicates greater cigarette dependence). The questionnaires are available as an appendix to this paper.

Statistical analysis

In order to quantify the proportion of quit attempts made after the first trimester of pregnancy, we aimed to recruit 850 participants.[24] Analyses were conducted using Stata version 14.0 (Stata Corp, College Station, TX, USA).

Descriptive statistics summarised participants' characteristics and smoking behaviour at each time point and we compared those responding to all three questionnaires with those who did not using chi-squared and t-tests for categorical and continuous variables respectively, with p values of <0.05 deemed significant. Characteristics found to be significantly associated with non-completion of later questionnaires, and hence absence of smoking data, were used with multiple imputation to adjust for attrition of smoking

For peer review only - http://bmjopen.bmj.com/site/about/guidelines.xhtml

behaviour at later time points.

An exploratory analysis was performed to investigate the factors associated with reporting having made a quit attempt of any duration on the baseline questionnaire. For this analysis items were dichotomised; six self-efficacy items that had high internal consistency (Cronbach's α=0.95)[27] were combined into a single score out of 30 with women scoring ≥ 25 considered to have high self-efficacy. Univariable logistic regression analysis was used to calculate an OR with 95% CIs for each variable (age,[28 29] ethnicity,[30] qualifications held,[28] previous pregnancy,[28 29 31 32] number of cigarettes smoked per day, HSI,[29 31 33] partner smoking status,[29] occupation,[34] planned or surprise pregnancy,[28 35-37] depression, long term disability or mental illness,[38] smoking beliefs and self-efficacy[39]). Variables which showed a significant association (p<0.05) in the univariable analysis were included in a multivariable logistic regression model. Variables that achieved significance (p<0.05) remained in the multivariable model and all non-significant variables identified from the univariable analysis were re-entered into the model consecutively to assess whether they became significant. The final multivariable model included only significant variables (p<0.05). A likelihood ratio test identified that age should be included in the multivariable analysis as a continuous variable. Where collinearity between variables was anticipated (for example, the number of cigarettes smoked per day and HSI), we included the variable that resulted in a better fitting model. As this analysis only included baseline data, we did not need to take account of attrition.

Multiple imputation for missing outcome data for smoking in late pregnancy (34-36 weeks gestation) and at 3 months after delivery was performed using Stata's mi command, based on 20 iterations. The outcomes were imputed using multivariable logistic regression models based on the following baseline variables: age, smoking

For peer review only

status, gestation, general health, depression, previous pregnancy, smoking in previous pregnancy, smoking urges, qualifications, and ethnicity. All baseline variables were included in the analysis in dichotomised format. The percentage of women smoking at each outcome was obtained using Rubin's rule.[40] Where necessary, an augmented regression approach was used to overcome issues relating to perfect prediction during the multiple imputation.

RESULTS

Figure 1 summarises questionnaire response rates. Of 1101 eligible women, 966 (88%) completed the baseline survey, and 850 (77%) consented to receive the later surveys. Questionnaires were returned by 509 (59.9%) in late pregnancy, and 476 (56.0%) at 3 months postpartum with 407 (47.8%) women completing all three.

[**Figure 1** Diagram showing recruitment and flow of participants through the study]

Participants had similar socio-demographic characteristics to those in previous pregnancy cohorts and have been reported elsewhere.[24] Just over half (488, 57.4%) were current smokers and 729 (85.7%) of the 850 women in the cohort reported their longer-term quitting intentions (data missing for 121 (14.2%)). Of these 729 women, 424 (58.2%) planned to stop smoking permanently, 21 (2.9%) intended to stop until their baby was born, and 181 (24.8%) were unsure; however, 103 (14.1%) did not plan to stop. Amongst the 272 smokers who reported a quit attempt at baseline, 14 (7.6%) reported stopping for >30 days, 32 (12.2%) for 7-30 days, 126 (48.1%) for 1-6 days, and 84 (32.1%) for <24 hours.

For peer review only

Responding to all three surveys was associated with being older, less cigarette dependant, primiparous, in a planned pregnancy and being a ‘recent ex-smoker’ at the outset of the study (Table 1).

For peer review only

Table 1 Comparison of participants who completed all three questionnaires with those who completed either one or two questionnaires.

**Characteristic**	**Completed all follow-up surveys N = 397 (46.7%)**	**Did not complete all surveys N = 453 (53.3%)**	**P value**
	**N (%)**	**N (%)**	
**Weeks gestation** (mean, SD)	15.8 (4.1)	15.4 (4.1)	0.14
**Age, years** (mean, SD)	26.5 (5.6)	25.2 (5.5)	<0.001**
**Baseline smoking status**			
Current smoker	199 (50.1)	289 (63.8)	
Ex-smoker	198 (49.9)	164 (36.2)	<0.001**
**Previous pregnancy**			
Not been pregnant before	143 (36.0)	132 (29.1)	
Been pregnant before	250 (63.0)	314 (69.3)	0.037*
**Partner smoking**			
Partner is not a current smoker/no partner	158 (39.8)	187 (41.3)	
Partner is a current smoker	236 (59.4)	263 (58.2)	0.67
**Current smokers only:**			
**Reported quit attempt since learning of pregnancy/previous questionnaire/birth of baby [a]**			
Yes	115 (57.8)	157 (54.3)	
No	78 (39.2)	122 (42.2)	0.47
**Heaviness of Smoking Index**			
Low dependence (0-2)	140 (70.4)	170 (58.8)	
Moderate dependence (3-4)	46 (23.1)	100 (34.6)	
High dependence (5-6)	1 (0.5)	7 (2.4)	0.004*

*significant at <0.05

**significant at <0.001

[a] Quit attempts: at baseline, since finding out about the pregnancy; at follow-up 1, since completing previous survey; follow-up 2, since birth of baby

For peer review only

Figure 2 shows a preliminary descriptive analysis of smoking behaviour across pregnancy within the 397 participants who returned all three questionnaires and illustrates variability in individual's smoking behaviour. Of note, 13.5% (5/37) of women who had stopped smoking in the 3 months before pregnancy were smoking again 3 months after childbirth, whereas 34.2% (55/161) of women who reported that they had quit after finding out they were pregnant had returned to smoking 3 months postpartum. As these data are not adjusted for non-response at follow-up, they may not be consistent with adjusted figures reported below.

[**Figure 2** Change in smoking behaviour between early pregnancy, late pregnancy and postpartum amongst respondents to all 3 questionnaires (N = 397)]

Table 2 shows findings from univariable and multivariable analyses that investigated factors associated with baseline current smokers having reported making a quit attempt earlier in pregnancy. As these analyses only used baseline data, adjustment for attrition was not needed. After the multivariable modelling, four factors were independently associated with reporting previous quit attempts at baseline: smoking fewer daily cigarettes, agreeing that smoking during pregnancy can seriously harm the baby, being primiparous and having a planned pregnancy.

For peer review only

Table 2 Univariable and multivariable associations with previous quit attempts in pregnancy at baseline

			Univariable Model		Multivariable Model	
**Variable**	**Current smokers in each response category N**	**Women who made a quit attempt N (row %)**	**Odds Ratio (95% confidence intervals)**	**P value**	**Odds Ratio (95% confidence intervals)**	**P value**
**Age, years**						
<20	97	64 (66.0)	1.00			
21-25	179	101 (56.4)	0.63 (0.37-1.07)			
26-30	123	57 (46.3)	0.42 (0.24-0.74)	0.026		
Over 31	86	48 (55.8)	0.64 (0.35-1.19)			
**Age, years Mean (SD)**	25.3(5.4)	24.9 (5.7)	0.97 (0.94-1.01)	0.11		
**General Health**						
Excellent	68	45 (66.2)	1.00			
Good	348	178 (51.2)	0.49 (0.28-0.87)			
Fair	68	47 (69.1)	0.95 (0.46-1.97)	0.016		
Poor	2	1 (50.0)	0.44 (0.03-7.5)			
**Qualifications held**						
None	121	50 (41.3)	1.00			
GCSEs or above	367	222 (60.5)	2.27 (1.49-3.46)	0.0001		
**Previous pregnancy**						
Yes	346	169 (48.8)	1.00		1.00	
No	137	101 (73.7)	3.17 (2.02-4.98)	<0.0001	2.20 (1.33-3.66)	0.0019
**Number of cigarettes smoked per day**						
≤5	191	136 (71.2)	1.00		1.00	
6-10	151	86 (57.0)	0.56 (0.36-0.88)	<0.0001	0.65 (0.39-1.07)	
≥11	131	45 (34.4)	0.22 (0.14-0.36)		0.28 (0.16-0.48)	<0.0001
**HSI**						
Low dependence	310	196 (63.2)	1.00			
Moderate dependence	146	61 (41.8)	0.43 (0.29-0.64)	<0.0001		
High dependence	8	2 (25.0)	0.19 (0.04-0.98)			
**Urge to smoke in last 24 hours**						

For peer review only

No urges	23	14 (60.9)	1.00			
Urges	447	251 (56.2)	0.97 (0.42-2.24)	0.95		
**Strength of urges to smoke in last 24 hours**						
No urges	31	16 (51.6)	1.00			
Weak	334	194 (58.1)	1.28 (0.60-2.70)	0.82		
Strong	103	59 (57.3)	1.26 (0.55-2.86)			
**Partner smoking status**						
Non-smoking partner	111	57 (51.4)	1.00			
Smoking partner	334	189 (56.6)	0.88 (0.57-1.37)	0.50		
No partner	41	26 (63.4)	1.38 (0.69-2.73)			
**Home Ownership**						
Rent/other	427	234 (54.8)	1.00	0.47		
Own home	57	35 (61.4)	1.23 (0.70-2.17)			
**Current employment**						
Not in current paid work	324	163 (50.3)	1.00	0.0005		
In current paid work	164	109 (66.5)	2.01 (1.35-2.99)			
**Usual occupation**						
Manual/not-applicable	351	194 (55.3)	1.00	0.13		
Non-manual	75	46 (61.3)	1.50 (0.88-2.57)			
**Ethnicity**						
White British	447	250 (55.9)	1.00			
Other	39	21 (53.9)	1.05 (0.53-2.10)	0.88		
**Timing of pregnancy**						
Planned	171	110 (64.3)	1.00		1.00	
Surprise	312	158 (50.6)	0.59 (0.40-0.87)	0.007	0.53 (0.34-0.82)	0.0045
**Felt depressed or hopeless in last month**						
Yes	144	88 (61.1)	1.00	0.21		
No	338	181 (53.6)	0.77 (0.52-1.16)			
**Long term disability or**						

For peer review only

**mental illness**						
Yes	66	34 (51.5)	1.00			
No	416	234 (56.3)	1.25 (0.74-2.11)	0.41		
**Smoking during pregnancy can harm your baby**						
Disagree	211	81 (38.4)	1.00		1.00	
Agree	266	183 (68.8)	4.08 (2.76-6.02)	<0.0001	4.23 (2.76-6.48)	<0.0001
**Self-efficacy in quitting**						
Low	412	220 (53.4)	1.00			
High	47	35 (74.5)	3.72 (1.68-8.21)	0.001		

For peer review only

At baseline, smokers reported making a median (IQR) of 2 (1-3) quit attempts lasting at least 24 hours since discovering they were pregnant; in later pregnancy 2 (1-5) quit attempts were reported since completing the first questionnaire and in the postpartum 2 (1-4) quit attempts were reported since childbirth (unadjusted data). The median number of quit attempts made by those who smoked across their pregnancy (smokers who completed both baseline and late pregnancy questionnaires, n=177) was 3 (IQR 1-6); these data were highly skewed with a range of 0-60 24-hour quit attempts reported.

Table 3 shows data on smoking rates, quitting behaviour and quit intentions at the three time points adjusted for non-response, as appropriate, using multiple imputation; raw (unadjusted) data are included for reference in a supplementary online table (Appendix Table S1). Adjusted figures show no evidence that smoking rates changed in pregnancy; the proportion of smokers was 57.4% (95% CI 54.1-60.7) at baseline and 59.1% (95% CI 54.9-63.4) in late pregnancy. However, by 3 months postnatally, the adjusted proportion of current smokers was 67.1% (95% CI 62.7-71.5). Over half (55.7%, 95% CI 51.3-60.1) of smokers reported making quit attempts since becoming pregnant and 51.3% (95% CI 44.7- 58.0) did so between early and late pregnancy; however, only 27.4% (95% CI 21.7-33.2) reported trying to stop after childbirth. The proportion of women who intended to try quitting within the next month fell as pregnancy progressed from 40.4% (95% CI 36.1-44.8) at baseline to 29.7% (95% CI 23.8-35.6) in late pregnancy and only 14.2% (95% CI 10.0-18.3) postpartum.

For peer review only

Table 3 Smoking behaviours reported in pregnancy and the postpartum adjusted for non-response at late pregnancy and 3 months postpartum using multiple imputation

Characteristic	Baseline (Early pregnancy)			Late pregnancy		Postpartum	
	n	%	95% CI	%	95% CI	%	95% CI
**Current smokers [a]**	488	57.4	54.1-60.7	59.1	54.9-63.4	67.1	62.7-71.5
**Reported quit attempt since learning of pregnancy/previous questionnaire/birth of baby [b]**							
Yes	272	55.7	51.3-60.1	51.3	44.7-58.0	27.4	21.7-33.2
No	200	41.0	36.7-45.4	48.7	42.0-55.3	72.6	66.8-78.3
**If have made a quit attempt, attempt lasted at least 24 hours**							
Yes	178	65.4	59.6-71.0	78.9	71.6-86.2	67.8	55.8-79.8
No	90	33.1	27.7-38.9	21.1	13.8-28.4	32.2	20.2-44.2
**Cigarettes per day**							
0-10	342	70.1	65.9-74.0	68.3	63.2-73.3	60.0	54.3-65.7
≥11	131	26.8	23.1-31.0	31.6	26.6-36.7	40.0	34.3-45.7
**How soon after waking smoke first cigarette**							
≤30 minutes	260	53.3	48.8-57.7	48.6	43.1-54.2	48.2	41.8-54.6
≥31 minutes	206	42.2	37.9-46.7	51.4	45.8-56.9	51.8	45.4-58.2
**Heaviness of Smoking Index**							
Low dependence (0-2)	310	63.5	59.1-67.7	68.1	62.5-73.8	65.7	59.1-72.3
Moderate-high dependence (3-6)	154	32.6	27.6-35.8	31.9	26.2-37.5	34.3	27.7-40.9
**Intention to quit smoking**							
Intending to quit within next 30 days	197	40.4	36.1-44.8	29.7	23.8-35.6	14.2	10.0-18.3
Intending to quit within next 3 months/not seriously planning to quit	252	51.6	47.2-56.1	70.3	64.4-76.2	85.8	81.7-90.0
**Urges to smoke**							
**How often felt urges to smoke in previous 24 hours**							
No/few urges/don't know	707	83.2	80.5-85.5	82.0	78.7-85.3	78.7	74.8-82.6
Frequent urges (a lot of the time-all of the time)	135	15.9	13.6-18.5	18.0	14.7-21.3	21.3	17.4-25.2
**Strength of urges to smoke in previous 24 hours**							
No urges/slight-moderate urges/don't know	738	86.8	84.4-88.9	82.8	79.0-86.5	83.0	79.0-87.0
Strong-extremely strong urges	109	12.8	10.7-15.2	17.2	13.5-21.0	17.0	13.0-21.0

[a] Includes those who report smoking regularly and those who smoke occasionally (not every day)

For peer review only

[b] Quit attempts: at baseline, since finding out about the pregnancy; at follow-up 1, since completing previous survey; follow-up 2, since birth of baby

Adjusted data show some differences in the rates of women restarting smoking according to when they report that they initially quit. Among women who had either not smoked in the 3 months before pregnancy or during early pregnancy (before completing the baseline questionnaire), 10.2% (95% CI 6.5-13.9) reported smoking on the late pregnancy questionnaire and 31.2% (95% CI 25.2-37.2) did so at 3 months postpartum. However, if this information is broken down further, for those who said they quit *prior* to becoming pregnant, only 2.5% reported smoking by late pregnancy and 17.8% (95% CI 6.1-29.5) reported smoking 3 months postpartum. Whereas, of those who reported quitting *after* finding out they were pregnant (but before completing the baseline questionnaire), 11.6% (95% CI 7.3-15.9) were smoking by late pregnancy, and 34.4% (95% CI 27.6-41.2) were smoking 3 months postpartum. By comparison, among smokers at baseline who reported not smoking in late pregnancy, 42.9% (95% CI 24.6-61.3) were smoking 3 months after delivery. Overall, of women who reported abstinence on the late pregnancy questionnaire, 26.2% (95% CI 20.3-32.2) had relapsed by 3 months post-delivery.

DISCUSSION

To our knowledge, this is the first study to use prospectively collected, longitudinal data to quantify changes in smoking behaviour through the examination of multiple quit attempts and women's intention to quit during pregnancy and postnatally. Despite over 50% of smokers reporting quit attempts across all three trimesters, there was no evidence that overall smoking rates changed between joining the study at around 8-24 weeks gestation and late pregnancy. In smokers, intention to quit within the next month fell as the pregnancy progressed, and then fell further postpartum. Within 3 months of

For peer review only

giving birth, around one third of women who achieved abstinence before or during early pregnancy had returned to smoking. However, we observed a trend, not previously reported in longitudinal data, whereby those who quit before pregnancy may be less likely to return to smoking postpartum than those that quit on learning of their pregnancy; those that only achieved abstinence in late pregnancy appeared to be most likely to return to smoking postpartum. Women's motivation to try quitting was lowest in the first 3 months following childbirth; only around a quarter tried quitting during this time and far fewer reported intending to quit in the immediate future than had done so at either pregnancy time point.

The originality of this study is a key strength. As previously mentioned, we could find relatively few observational studies in which pregnant women's smoking behaviours were longitudinally recorded at more than one time point in pregnancy and .[12 13 15 16 19-23] Only two of these longitudinal studies followed women up postpartum,[12 13] only four reported any data on fluctuations or trajectories in smoking status,[12 13 15 16] and none evaluated multiple quit attempts, often assessing only heaviness of smoking, or successful versus unsuccessful quitting. All other studies investigating individuals' changes in smoking behaviour in pregnancy have asked about this retrospectively after pregnancy or at only one time point, and in contrast, we collected longitudinal data during and after pregnancy. We particularly focussed on quit attempts and quitting intentions, rather than purely on smoking status at different time points, and are not aware of any other longitudinal studies that have attempted this. Additionally, for the first time we have reported 'attrition-adjusted' rates of smoking or quitting in later pregnancy and postpartum. If we had only used cross-sectional data, we might have underestimated the proportion of smokers in later pregnancy and in the postpartum. We believe that we present the most robust available data documenting changes in smoking status and quitting behaviours across pregnancy and into postpartum, for women who are not participating in an intervention study.

For peer review only

A limitation is that, although we followed women longitudinally, at each data collection point we relied on self-reported data and recall of smoking behaviour in the immediate past, so we cannot be completely sure that reports are valid. However, a number of factors should have minimised any misreporting of smoking behaviour: no intervention was tested and there was no expectation that participants should try stopping; researchers emphasised that responses were of interest irrespective of smoking status and, as women completed questionnaires at three stages, they did not have to recollect their behaviour over long periods. Additionally, studies looking at both self-report and biochemically-validated smoking data suggest that self-reported smoking can be both accurate and reliable.[16 41] It is possible that pregnant women who were concerned about the stigma of smoking may have avoided participation; we do not know how this might have affected findings, but women who consented to join the cohort had similar characteristics to those who declined.[24] As the survey was conducted in just two Nottingham hospitals, it is hard to say how far findings can be generalised. To help assess generalisability, we included survey items that permitted comparison with previous studies; we found our participants who continued to smoke in early pregnancy were similar to pregnant smokers enrolled in other major UK cohorts.[24] This suggests that the principal findings may apply to pregnant smokers in the UK generally. Likewise, although absolute smoking rates and smoking cessation advice and treatment may vary, pregnant smokers from other high income countries generally have similar characteristics to those in the UK.[28 42 43] Therefore, it could be considered reasonable to extrapolate many of our findings to pregnant smokers in high income countries generally. Although we had very high rates of eligible women joining the cohort, a further limitation was that attrition was relatively high, with response rates to the two later questionnaires of 60% and 56%. This is a common problem with longitudinal studies,[44] and as young, pregnant smokers were likely to be a particularly difficult group to maintain contact with, we used a number of recommended methods to try to

For peer review only

maximise response rates.[24 44 45] However, rather than simply relying on incomplete data, we have tried to address non-response bias by adjusting later surveys' findings using multiple imputation. In addition, differences in characteristics between the whole cohort and those that responded to all three surveys (Table 1) need to be considered when viewing the unadjusted smoking 'trajectory' analysis shown in Figure 2. Finally, we assessed smoking status at 3 months postpartum, and it is likely that some women who were abstinent at this point will have returned to smoking after this.[17 18]

The finding that most women in our cohort had quit in the early stages of pregnancy (before joining the study), and that smoking rates did not change between the second trimester and 36 weeks' gestation is consistent with cross-sectional estimates for smoking prevalence obtained in a large US study, which reported these by month of pregnancy.[46] In that study, smoking prevalence at 1 month gestation was 26%, then between the fourth and eighth month of gestation, smoking rates each month were 13-14%.[46] Other retrospective studies have found that most women who successfully quit are likely to achieve this soon after finding out they are pregnant,[5 47] often within the first few days.[47] Many quit spontaneously after discovering they are pregnant.[48] Therefore, it seems that after the early stages of pregnancy, despite still reporting quit attempts, women's smoking behaviour actually undergoes very little change.

One study found that 70% of pregnant women making their first quit attempt did so in their first trimester; however, these data were collected up to 5 years after delivery, and only considered first quit attempts.[15] We found that some women made multiple quit attempts throughout pregnancy and we have previously reported that, at baseline, most reported cutting down or only smoking occasionally since becoming pregnant with less than 8% of our cohort saying that they smoked the same or more than before pregnancy.[24] Although self-reported, this reinforces findings from qualitative studies,

For peer review only

which indicate that many persistent smokers report deliberate, and sometimes detailed, plans to cut down in their pregnancy, seeing this as a positive step and often as a route to quitting.[49] Far fewer women reported making quit attempts in the 3 months after childbirth than they did during pregnancy. Even in early pregnancy, around half of women had no intention to quit within the next 30 days; intention to quit in the short term was even lower in late pregnancy, and was lowest of all postpartum. This diminishing intention to quit has not been reported before and could be considered when designing and delivering cessation interventions; for example, earlier intervention may be more successful.

We found that women who were primiparous, smoked fewer cigarettes per day, had a planned pregnancy and believed smoking during pregnancy could seriously harm their baby were more likely to have made a quit attempt during early pregnancy. These findings are comparable to previous literature examining the characteristics of pregnant smokers who successfully achieve cessation. Primiparous women have previously been found to be more likely to successfully quit smoking.[28 29 31 32] This may be because women who have smoked throughout a previous pregnancy without experiencing complications may view the risks of smoking during pregnancy differently to primiparous women, and therefore be less motivated to make a quit attempt.[29] Similarly, previous studies have found that heavier smoking is negatively associated with successfully quitting in pregnancy,[29 31 33] and heavier smokers are less likely to have high motivation to quit during pregnancy.[33] Women whose pregnancies are unintended have previously been found to be more likely to continue smoking during pregnancy,[28 35-37] and likewise pregnant smokers who do not report concern about the effect smoking might have on the health of their unborn baby were more likely to have low motivation to quit smoking.[33] These findings identify women who are most likely to make a quit attempt and will potentially benefit the most from NHS support. Heavier smokers and women in second or later and unplanned pregnancies who are less likely to try quitting may require

For peer review only

different, more intensive or tailored forms of support.

Implications for practice

Although our data suggest that motivation to quit may be strongest in early pregnancy, some women will be receptive to quitting at any time, as indicated by their multiple quit attempts throughout pregnancy, and this confirms that it is important to discuss smoking with women at every appointment and to refer them for stop smoking support.[9] One rather surprising finding was that in early to mid-pregnancy 44% (211/477) of smokers disagreed that smoking in pregnancy can harm their baby; as those who agreed with this statement were more likely to have made previous quit attempts at baseline, additional education on this issue should be considered by health professionals. Preventing resumption of smoking after pregnancy is a critical public health issue; if women restart their lifelong health is at risk, and their infants are more likely to be exposed to second-hand smoke[50] and to eventually become smokers.[51] Women often need help to resist returning to smoking after childbirth, but there are currently few effective interventions for this.[52] Women appear to be more inclined to consider quitting during pregnancy than in postpartum, and this is important when designing interventions. A potential reason for restarting smoking and for making fewer quit attempts postpartum may be that women perceive that harm to the baby from smoking is much higher during pregnancy compared with after delivery. However, some postpartum women do make quit attempts, or may be planning to quit in the medium term, so engaging with them again after birth, to think about planning for this in the medium term, rather than immediately, might be a successful option. Previous studies have shown that women who quit spontaneously early in pregnancy are likely to be different and more successful than those who quit later,[48] and we found that women appear to be more likely to return to smoking after childbirth the later in pregnancy they quit. Therefore, exploring potential reasons for this, for example demographic factors or

For peer review only

women's intentions, could help to identify if different women may benefit from alternative approaches to help prevent relapse, perhaps by developing more tailored interventions. Although quit attempts might suggest receptivity to quitting, what is not well understood is how interest in smoking cessation support may change during pregnancy.

Conclusions

Many pregnant women who smoke attempt quitting throughout their pregnancy, but this makes little difference to overall smoking rates. After giving birth, most smokers seem less inclined to make further quit attempts and many who quit in early pregnancy return to smoking. Women who quit in late pregnancy may be most likely to return to smoking after childbirth, whilst those who stopped prior to pregnancy may be least likely to relapse. It therefore imperative to discuss smoking with women, including recent ex-smokers, throughout pregnancy and postpartum, and to continue to offer and provide specialist stop smoking support.

For peer review only

ACKNOWLEDGEMENTS

The authors would like to thank all participants and staff involved in this study, and Nottingham University Hospitals NHS Trust for facilitating this research. We also thank James Brimicombe for developing the study database, and Rachel Whitemore for her invaluable help with this study.

CONTRIBUTORS

SC helped conceive the study, made a substantial contribution to the development of the protocol and questionnaires, assisted with day-to-day troubleshooting during the data-collection phase, and drafted and revised this manuscript. SO helped design the data collection process, recruited participants into the cohort, managed the day-to-day running, assisted with data analysis, and contributed to the drafting of this manuscript. JL-B contributed to the development of the study protocol and questionnaires, advised on analysis, and contributed to the preparation of this manuscript. EB undertook the analyses and interpretation of data in Table 2 as part of a BMedSci project and contributed to the preparation of this manuscript. LV assisted with data analysis and interpretation, and contributed to the preparation of this manuscript. KB helped design the data collection process, recruited participants into the cohort, managed the day-to-day running, and contributed to the preparation of this manuscript. FN, MU, KEP and SS all contributed to the development of the study protocol and questionnaires, contributing expertise from their own particular knowledge base, and to the preparation of this manuscript. TC conceived the study, and made substantial contributions to the development of the study protocol and questionnaires, and to the preparation of this manuscript. All authors read and approved the final manuscript. Rachel Whitemore assisted with study administration, telephone follow-ups, and data entry.

For peer review only

FUNDING

This article presents independent research funded by the National Institute for Health Research (NIHR) under its Programme Grants for Applied Research Programme (Grant Number RP-PG 0109-10020). The views expressed in this paper are those of the authors and not necessarily those of the NHS, the NIHR or the Department of Health.

SC, SO, JLB, KB, FN, MU, SS and TC are members of the UK Centre for Tobacco and Alcohol Studies (UKCTAS), a UKCRC Public Health Research Centre of Excellence. UKCTAS receives core funding from the British Heart Foundation, Cancer Research UK, Economic and Social Research Council, Medical Research Council and the Department of Health under the auspices of the UK Clinical Research Collaboration. SC, SO, KB, SS and TC are members of the NIHR School for Primary Care Research. TC acknowledges the support of the National Institute for Health Research (NIHR) Collaboration for Leadership in Applied Health Research.

COMPETING INTERESTS

KP is a trustee of The Equality Trust (a registered charity), and receives occasional honoraria, all of which are donated to The Equality Trust or for student support at the University of York. In the last 5 years TC has been paid honoraria on 2 occasions for speaking at meetings or conferences organised by Pierre Fabre Laboratories (a nicotine replacement therapy manufacturer).

Other authors have no conflicts of interest to report.

ETHICS APPROVAL

Derbyshire Research Ethics Proportionate Review Sub-Committee gave ethical approval.

For peer review only

PROVENANCE AND PEER REVIEW

Not commissioned; externally peer reviewed.

DATA SHARING STATEMENT

The dataset is still subject to further analyses. Relevant anonymised data available from the authors on reasonable request.

For peer review only

REFERENCES

1. British Medical Association. Smoking and reproductive life: The impact of smoking on sexual, reproductive and child health. London: BMA, 2004.

2. Cnattingius S. The epidemiology of smoking during pregnancy: smoking prevalence, maternal characteristics, and pregnancy outcomes. *Nicotine Tob Res* 2004;6(S2):S125-S40. doi: 10.1080/14622200410001669187

3. Jensen KE, Jensen A, Nohr B, et al. Do pregnant women still smoke? A study of smoking patterns among 261,029 primiparous women in Denmark 1997-2005. *Acta Obstet Gynecol Scand* 2008;87(7):760-67. doi: Doi 10.1080/00016340802179814

4. Mohsin M, Bauman AE, Forero R. Socioeconomic correlates and trends in smoking in pregnancy in New South Wales, Australia. *J Epidemiol Community Health* 2011;65(8):727-32. doi: 10.1136/jech.2009.104232 [published Online First: 2010/09/16]

5. McAndrew F, Thompson J, Fellows L, et al. Infant Feeding Survey 2010: Health and Social Care Information Centre, 2012.

6. Tong VT, Dietz PM, Morrow B, et al. Trends in smoking before, during, and after pregnancy--Pregnancy Risk Assessment Monitoring System, United States, 40 sites, 2000-2010. *MMWR Surveill Summ* 2013;62(6):1-19. [published Online First: 2013/12/19]

7. Bloch M, Althabe F, Onyamboko M, et al. Tobacco use and secondhand smoke exposure during pregnancy: An investigative survey of women in 9 developing nations. *Am J Public Health* 2008;98(10):1833-40. doi: Doi 10.2105/Ajph.2007.117887

8. Fahy SJ, Cooper S, Coleman T, et al. Provision of smoking cessation support for pregnant women in England: results from an online survey of NHS stop smoking services for pregnant women. *BMC Health Serv Res* 2014;14(1):107. doi: 10.1186/1472-6963-14-107

9. National Institute for Health and Clinical Excellence. Public health guidance 26: How to stop smoking in pregnancy and following childbirth. London, 2010.

10. Chassin L, Presson CC, Pitts SC, et al. The natural history of cigarette smoking from adolescence to adulthood in a midwestern community sample: multiple trajectories and their psychosocial correlates. *Health Psychol* 2000;19(3):223-31. [published Online First: 2000/06/27]

11. Fitzpatrick KE, Gray R, Quigley MA. Women's Longitudinal Patterns of Smoking during the Pre-Conception, Pregnancy and Postnatal Period: Evidence from the UK Infant Feeding Survey. *PLoS One* 2016;11(4):e0153447. doi: 10.1371/journal.pone.0153447

12. Munafo MR, Heron J, Araya R. Smoking patterns during pregnancy and postnatal period

For peer review only

and depressive symptoms. *Nicotine Tob Res* 2008;10(11):1609-20.

13. Eiden RD, Homish GG, Colder CR, et al. Changes in Smoking Patterns During Pregnancy. *Subst Use Misuse* 2013;48(7):513-22. doi: doi:10.3109/10826084.2013.787091

14. Mumford EA, Hair EC, Yu T-C, et al. Women's Longitudinal Smoking Patterns from Preconception Through Child's Kindergarten Entry: Profiles of Biological Mothers of a 2001 US Birth Cohort. *Matern Child Health J* 2014;18(4):810-20. doi: 10.1007/s10995-013-1305-y

15. Pickett KE, Wakschlag LS, Dai L, et al. Fluctuations of maternal smoking during pregnancy. *Obstet Gynecol* 2003;101(1):140-47.

16. Pickett KE, Rathouz PJ, Kasza K, et al. Self-reported smoking, cotinine levels, and patterns of smoking in pregnancy. *Paediatr Perinat Epidemiol* 2005;19(5):368-76.

17. Fingerhut LA, Kleinman JC, Kendrick JS. Smoking before, during, and after pregnancy. *Am J Public Health* 1990;80(5):541-44.

18. Jones M, Lewis S, Parrott S, et al. Re-starting smoking in the postpartum period after receiving a smoking cessation intervention: a systematic review. *Addiction* 2016;111:981-90. DOI: 10.1111/add.13309. doi: DOI: 10.1111/add.13309

19. Appleton PL, Pharoah POD. Partner smoking behaviour change is associated with women's smoking reduction and cessation during pregnancy. *Br J Health Psychol* 1998;3(4):361-74. doi: 10.1111/j.2044-8287.1998.tb00580.x

20. Wakschlag LS, Pickett KE, Middlecamp MK, et al. Pregnant smokers who quit, pregnant smokers who don't: does history of problem behavior make a difference? *Soc Sci Med* 2003;56(12):2449-60. doi: http://dx.doi.org/10.1016/S0277-9536(02)00248-4

21. Espy KA, Fang H, Johnson C, et al. Prenatal Tobacco Exposure: Developmental Outcomes in the Neonatal Period. *Dev Psychol* 2011;47(1):153-56. doi: 10.1037/a0020724

22. Maxson PJ, Edwards SE, Ingram A, et al. Psychosocial differences between smokers and non-smokers during pregnancy. *Addict Behav* 2012;37(2):153-59. doi: http://dx.doi.org/10.1016/j.addbeh.2011.08.011

23. Massey SH, Estabrook R, O'Brien TC, et al. Preliminary Evidence for the Interaction of the Oxytocin Receptor Gene (OXTR) and Face Processing in Differentiating Prenatal Smoking Patterns. *Neurosci Lett* 2015;0:259-64. doi: 10.1016/j.neulet.2014.10.049

24. Orton S, Bowker K, Cooper S, et al. Longitudinal cohort survey of women's smoking behaviour and attitudes in pregnancy: study methods and baseline data. *BMJ Open* 2014;4(5):e004915. doi: 10.1136/bmjopen-2014-004915

25. von Elm E, Altman DG, Egger M, et al. The Strengthening the Reporting of

For peer review only

Observational Studies in Epidemiology (STROBE) statement: guidelines for reporting observational studies. *Lancet* 2007;370(9596):1453-57. doi: 10.1016/s0140-6736(07)61602-x

26. Heatherton TF, Kozlowski LT, Frecker RC, et al. Measuring the heaviness of smoking: using self-reported time to the first cigarette of the day and number of cigarettes smoked per day. *Br J Addict* 1989;84(7):791-99.

27. Cronbach LJ. Coefficient alpha and the internal structure of tests. *Psychometrika* 1951;16(3):297-334. doi: 10.1007/bf02310555

28. Smedberg J, Lupattelli A, Mårdby AC, et al. Characteristics of women who continue smoking during pregnancy: A cross-sectional study of pregnant women and new mothers in 15 European countries. *BMC Pregnancy Childbirth* 2014;14:213. doi: 10.1186/1471-2393-14-213

29. Giglia RC, Binns CW, Alfonso HS. Which women stop smoking during pregnancy and the effect on breastfeeding duration. *BMC Public Health* 2006;6:195. doi: 10.1186/1471-2458-6-195

30. Tong VJ, JR.; Dietz, PM.; D'Angelo, D.; Bombard, JM. Trends in Smoking Before, During, and After Pregnancy — Pregnancy Risk Assessment Monitoring System (PRAMS), United States, 31 Sites, 2000–2005. *MMWR Surveill Summ* 2009;58(SS-4):1-36.

31. Hakansson A, Lendahls L, Petersson C. Which women stop smoking? A population-based study of 403 pregnant smokers. *Acta Obstet Gynecol Scand* 1999;78(3):217-24.

32. Lindqvist R, Aberg H. Who stops smoking during pregnancy? *Acta Obstet Gynecol Scand* 2001;80(2):137-41.

33. Ershoff DH, Solomon LJ, Dolan-Mullen P. Predictors of intentions to stop smoking early in prenatal care. *Tob Control* 2000;9 Suppl 3:III41-5.

34. Graham H, Hawkins SS, Law C. Lifecourse influences on women's smoking before, during and after pregnancy. *Soc Sci Med* 2010;70(4):582-87. doi: http://dx.doi.org/10.1016/j.socscimed.2009.10.041

35. Dott M, Rasmussen SA, Hogue CJ, et al. Association Between Pregnancy Intention and Reproductive-health Related Behaviors Before and After Pregnancy Recognition, National Birth Defects Prevention Study, 1997–2002. *Matern Child Health J* 2010;14(3):373-81. doi: 10.1007/s10995-009-0458-1

36. McCrory C, McNally S. The effect of pregnancy intention on maternal prenatal behaviours and parent and child health: results of an irish cohort study. *Paediatr Perinat Epidemiol* 2013;27(2):208-15. doi: 10.1111/ppe.12027

37. Chisolm MS, Cheng D, Terplan M. The relationship between pregnancy intention and

For peer review only

change in perinatal cigarette smoking: an analysis of PRAMS data. *J Subst Abuse Treat* 2014;46(2):189-93. doi: 10.1016/j.jsat.2013.07.010

38. McCormick MC, Brooks-Gunn J, Shorter T, et al. Factors associated with smoking in low-income pregnant women: relationship to birth weight, stressful life events, social support, health behaviors and mental distress. *J Clin Epidemiol* 1990;43(5):441-48.

39. Devries H, Backbier E. Self-efficacy as an important determinant of quitting among pregnant women who smoke: the Ø-pattern. *Prev Med* 1994;23(2):167-74.

40. Rubin DB. Multiple imputation for nonresponse in surveys. New York: John Wiley and Sons 1987.

41. George L, Granath F, Johansson ALV, et al. Self-reported nicotine exposure and plasma levels of cotinine in early and late pregnancy. *Acta Obstet Gynecol Scand* 2006;85(11):1331-37.

42. Lu Y, Tong S, Oldenburg B. Determinants of smoking and cessation during and after pregnancy. *Health Promot Int* 2001;16(4):355-65. doi: 10.1093/heapro/16.4.355

43. Schneider S, Schütz J. Who smokes during pregnancy? A systematic literature review of population-based surveys conducted in developed countries between 1997 and 2006. *Eur J Contracept Reprod Health Care* 2008;13(2):138-47. doi: 10.1080/13625180802027993

44. Ribisl KM, Walton MA, Mowbray CT, et al. Minimizing participant attrition in panel studies through the use of effective retention and tracking strategies: Review and recommendations. *Eval Program Plann* 1996;19(1):1-25. doi: http://dx.doi.org/10.1016/0149-7189(95)00037-2

45. Edwards P, Roberts I, Clarke M, et al. Methods to increase response rates to postal questionnaires. *Cochrane Database Syst Rev* 2007(2):MR000008.

46. Alshaarawy O, Anthony JC. Month-Wise Estimates of Tobacco Smoking During Pregnancy for the United States, 2002–2009. *Matern Child Health J* 2015;19(5):1010-15. doi: 10.1007/s10995-014-1599-4

47. Heil SH, Herrmann ES, Badger GJ, et al. Examining the timing of changes in cigarette smoking upon learning of pregnancy. *Prev Med* 2014;68:58-61. doi: http://dx.doi.org/10.1016/j.ypmed.2014.06.034

48. Solomon LJ, Quinn VP. Spontaneous quitting: Self-initiated smoking cessation in early pregnancy. *Nicotine Tob Res* 2004;6(SUPPL. 2):S203-S16. doi: 10.1080/14622200410001669132

49. Graham H, Flemming K, Fox D, et al. Cutting down: insights from qualitative studies of smoking in pregnancy. *Health & Social Care in the Community* 2014;22(3):259-67. doi:

For peer review only

10.1111/hsc.12080
50. Orton S, Jones LL, Cooper S, et al. Predictors of Children's Secondhand Smoke Exposure at Home: A Systematic Review and Narrative Synthesis of the Evidence. *PLoS One* 2014;9(11):e112690. doi: 10.1371/journal.pone.0112690
51. Leonardi-Bee J, Jere ML, Britton J. Exposure to parental and sibling smoking and the risk of smoking uptake in childhood and adolescence: a systematic review and meta-analysis. *Thorax* 2011;66(10):847-55. doi: 10.1136/thx.2010.153379
52. Hajek P, Stead Lindsay F, West R, et al. Relapse prevention interventions for smoking cessation. *Cochrane Database Syst Rev* 2013(8):CD003999. doi: 10.1002/14651858.CD003999.pub4

For peer review only

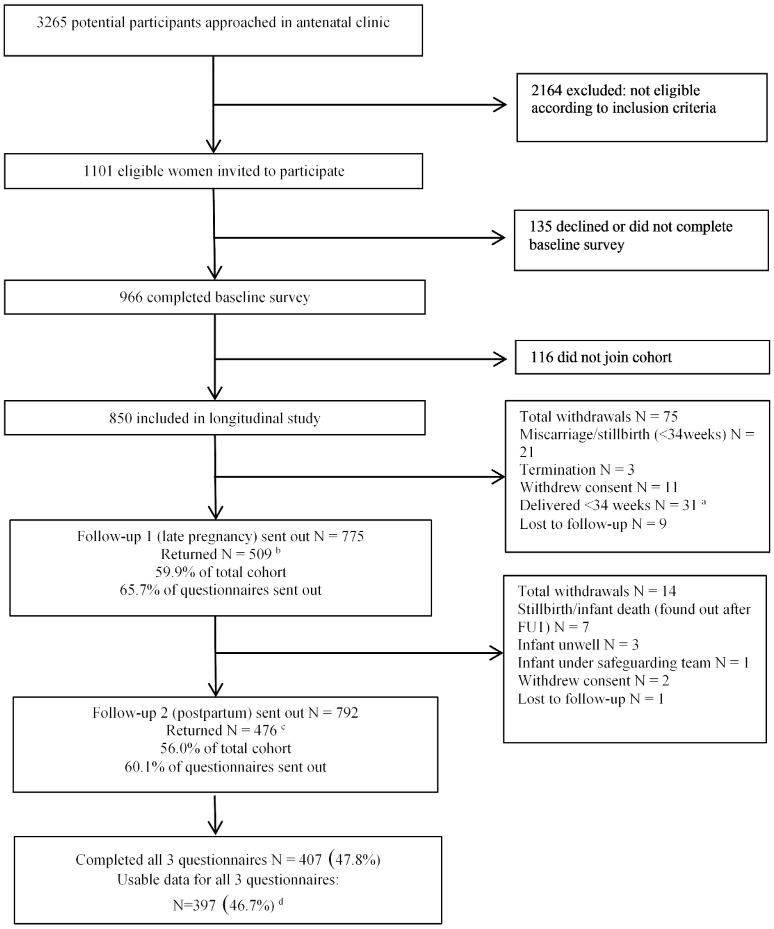

[a] 28 of the participants who delivered before 34 weeks gestation were sent the postpartum questionnaire

[b] 13 excluded who completed a follow-up 1 questionnaire, but weren't eligible (i.e. any women who was not still pregnant at 34 weeks)

[c] 70 participants who did not complete a late pregnancy questionnaire returned the postpartum questionnaire; of these, seven were women who delivered before 34 weeks gestation

[d] 10 excluded who completed all 3 questionnaires, but weren't eligible to complete follow-up 1 questionnaire (i.e. if miscarriage/stillbirth before 34 weeks gestation, terminated pregnancy or gave birth before 34 weeks)

Figure 1 Diagram showing recruitment and flow of participants through the study

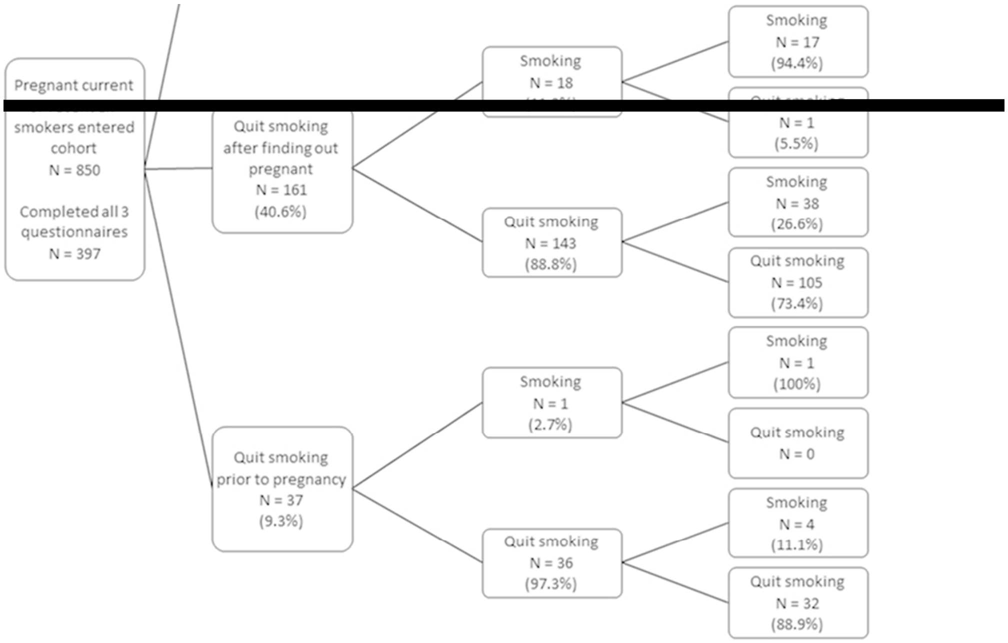

Figure 2 Change in smoking behaviour between early pregnancy, late pregnancy and postpartum amongst respondents to all 3 questionnaires (N = 397)

only

Appendix Table S1 Cross-sectional data: participants' smoking behaviour for returned questionnaires only at each follow up point

Characteristic	Baseline (Early pregnancy) N (%)	Late pregnancy N (%)	Postpartum N (%)
**Respondents** (response rate)	850	509 (59.9)	476 (56.0)
**Smoking status**			
Current smoker	488 (57.4)	252 (49.5)	281 (59.0)
Recent ex-smoker	362 (42.6)	257 (50.5)	195 (41.0)
**Current smoking behaviour (all participants)**			
Stopped smoking during 3 months prior to pregnancy	61 (7.2)		
Stopped smoking after learning of pregnancy/don't smoke at all now	301 (35.4)	257 (50.5)	195 (41.0)
Smoke occasionally, but not every day now pregnant	117 (13.8)	61 (12.0)	84 (17.6)
Smoke every day, but have cut down since: learning of pregnancy/during pregnancy	304 (35.8)	144 (28.3)	56 (11.8)
Smoke every day, about the same as before/during pregnancy	64 (7.5)	42 (8.3)	80 (16.8)
Smoke every day, tend to smoke more than before/during pregnancy	3 (0.4)	4 (0.8)	59 (12.4)
Missing	0	1 (0.2)	2 (0.4)
**Current smokers only:**			
**Reported quit attempt since learning of pregnancy/previous questionnaire/birth of baby** [a]			
Yes	272 (55.7)	130 (51.6)	78 (27.8)
No	200 (41.0)	119 (47.2)	191 (68.0)
Missing	16 (3.3)	3 (1.2)	12 (4.3)
**If have made a quit attempt, attempt lasted at least 24 hours**			
Yes<br>(% of all current smokers)<br>(% of current smokers making a quit attempt)	178<br>(36.5%)<br>(65.4%)	103<br>(40.9%)<br>(79.2%)	61<br>(21.7%)<br>(78.2%)
No<br>(% of all current smokers)<br>(% of current smokers making a quit attempt)	90<br>(18.4%)<br>(33.1%)	19<br>(7.5%)<br>(14.6%)	6<br>(2.1%)<br>(7.7%)
**Number of quit attempts >24 hours, median (IQR)**	2 (1-3)	2 (1-5)	2 (1-4)
**Cigarettes per day**			
0-5	191 (39.1)	99 (39.3)	102 (36.3)
6-10	151 (30.9)	85 (33.7)	83 (29.5)
11-15	74 (15.2)	32 (12.7)	55 (19.6)
16-20	47 (9.6)	24 (9.5)	30 (10.7)
21-30	8 (1.6)	9 (3.6)	5 (1.8)
≥31	2 (0.4)	0 (0)	1 (0.4)
Missing	15 (3.1)	3 (1.2)	5 (1.8)
**Time to first cigarette**			
< 5 minutes	97 (19.9)	31 (12.3)	28 (10.0)
6-30 minutes	163 (33.4)	79 (31.3)	83 (29.5)
31-60 minutes	89 (18.2)	44 (17.5)	50 (17.8)

For peer review only

STROBE 2007 (v4) Statement—Checklist of items that should be included in reports of *cohort studies*

Section/Topic	Item #	Recommendation	Reported on page #
**Title and abstract**	1	(*a*) Indicate the study's design with a commonly used term in the title or the abstract	1
		(*b*) Provide in the abstract an informative and balanced summary of what was done and what was found	2
**Introduction**			
Background/rationale	2	Explain the scientific background and rationale for the investigation being reported	5
Objectives	3	State specific objectives, including any prespecified hypotheses	5
**Methods**			
Study design	4	Present key elements of study design early in the paper	6
Setting	5	Describe the setting, locations, and relevant dates, including periods of recruitment, exposure, follow-up, and data collection	6
Participants	6	(*a*) Give the eligibility criteria, and the sources and methods of selection of participants. Describe methods of follow-up	6
		(*b*) For matched studies, give matching criteria and number of exposed and unexposed	
Variables	7	Clearly define all outcomes, exposures, predictors, potential confounders, and effect modifiers. Give diagnostic criteria, if applicable	6-7
Data sources/ measurement	8*	For each variable of interest, give sources of data and details of methods of assessment (measurement). Describe comparability of assessment methods if there is more than one group	6-7
Bias	9	Describe any efforts to address potential sources of bias	7-8
Study size	10	Explain how the study size was arrived at	Previously described in methods paper
Quantitative variables	11	Explain how quantitative variables were handled in the analyses. If applicable, describe which groupings were chosen and why	7-8
Statistical methods	12	(*a*) Describe all statistical methods, including those used to control for confounding	7-8
		(*b*) Describe any methods used to examine subgroups and interactions	n/a
		(*c*) Explain how missing data were addressed	8
		(*d*) If applicable, explain how loss to follow-up was addressed	8
		(*e*) Describe any sensitivity analyses	n/a

For peer review only

**Results**			
Participants	13*	(a) Report numbers of individuals at each stage of study—eg numbers potentially eligible, examined for eligibility, confirmed eligible, included in the study, completing follow-up, and analysed	9 & Figure 1
		(b) Give reasons for non-participation at each stage	Figure 1
		(c) Consider use of a flow diagram	Figure 1
Descriptive data	14*	(a) Give characteristics of study participants (eg demographic, clinical, social) and information on exposures and potential confounders	9, Table 1, Appendix Table S1
		(b) Indicate number of participants with missing data for each variable of interest	Appendix Table S1
		(c) Summarise follow-up time (eg, average and total amount)	n/a
Outcome data	15*	Report numbers of outcome events or summary measures over time	
Main results	16	(*a*) Give unadjusted estimates and, if applicable, confounder-adjusted estimates and their precision (eg, 95% confidence interval). Make clear which confounders were adjusted for and why they were included	Throughout
		(*b*) Report category boundaries when continuous variables were categorized	Throughout
		(*c*) If relevant, consider translating estimates of relative risk into absolute risk for a meaningful time period	n/a
Other analyses	17	Report other analyses done—eg analyses of subgroups and interactions, and sensitivity analyses	
**Discussion**			
Key results	18	Summarise key results with reference to study objectives	17
**Limitations**			
Interpretation	20	Give a cautious overall interpretation of results considering objectives, limitations, multiplicity of analyses, results from similar studies, and other relevant evidence	18-21
Generalisability	21	Discuss the generalisability (external validity) of the study results	18
**Other information**			
Funding	22	Give the source of funding and the role of the funders for the present study and, if applicable, for the original study on which the present article is based	24

*Give information separately for cases and controls in case-control studies and, if applicable, for exposed and unexposed groups in cohort and cross-sectional studies.

Note: An Explanation and Elaboration article discusses each checklist item and gives methodological background and published examples of transparent reporting. The STROBE checklist is best used in conjunction with this article (freely available on the Web sites of PLoS Medicine at http://www.plosmedicine.org/, Annals of Internal Medicine at http://www.annals.org/, and Epidemiology at http://www.epidem.com/). Information on the STROBE Initiative is available at www.strobe-statement.org.

For peer review only
